# Time-resolved coherent X-ray scattering analysis of nanorod colloids: dynamics and photon-statistical effects in angular X-ray cross-correlation analysis

**DOI:** 10.1107/S160057752600980X

**Published:** 2026-10-27

**Authors:** Taiki Hoshino, Yoshifumi Amamoto, Takahiro Mikami, Yoshiaki Honjo, Kyosuke Ozaki, Toshiyuki Nishiyama Hiraki, Haruki Nishino, Yasumasa Joti, Takaki Hatsui, Yoshiki Kohmura, Takashi Kato

**Affiliations:** a International Center for Synchrotron Radiation Innovation Smart (SRIS) Tohoku University 468-1 Aramaki Aza-Aoba Aoba-ku Sendai980-8572 Japan; b Institute of Multidisciplinary Research for Advanced Materials Tohoku University 2-1-1 Katahira Aoba-ku Sendai980-8572 Japan; c RIKEN SPring-8 Center, 1-1-1 Kouto, Sayo-cho, Sayo-gun, Hyogo679-5148, Japan; d Graduate School of Frontier Sciences The University of Tokyo 5-1-5 Kashiwa-no-ha Kashiwa City Chiba277-8568 Japan; e Department of Chemistry and Biotechnology, School of Engineering The University of Tokyo 7-3-1 Hongo Bunkyo-ku Tokyo113-8656 Japan; f Japan Synchrotron Radiation Research Institute Kouto Sayo-cho. Sayo-gun Hyogo679-5198 Japan; g Institute for Aqua Regeneration Shinshu University 3-1-1 Asahi Matsumoto Nagano380-8621 Japan; h Research Institute for Interdisciplinary Science Okayama University 3-1-1 Tsushima-naka Kita-ku Okayama700-8530 Japan; European XFEL, Germany

**Keywords:** X-ray photon correlation spectroscopy, angular X-ray cross correlation function, coherent X-ray scattering, orientational fluctuations, photon statistics

## Abstract

Understanding the dynamic fluctuations of anisotropic particles is essential for elucidating the mechanisms of order formation through self-organization. Because anisotropic particles possess both translational and rotational degrees of freedom, their dynamics are intrinsically more complex than those of spherical particles. Time-resolved coherent X-ray scattering has the potential to probe such complex dynamics. Translational motion can be evaluated by X-ray photon correlation spectroscopy (XPCS), whereas orientational information can be extracted using the angular X-ray cross-correlation function (AXCCF). In this study, we investigated dynamic fluctuations in a colloidal nanorod system at concentrations near the coexistence region of isotropic and liquid-crystalline phases using time-resolved coherent X-ray scattering at 17.4 kHz. XPCS analysis revealed a pronounced slowing down of translational motion as the isotropic phase approached the liquid-crystalline phase. To examine possible temporal fluctuations in orientational order near the coexistence region, we applied a statistical approach based on AXCCF and principal component analysis. Temporal variations were indeed observed in the AXCCF. However, quantitative analysis showed that the magnitude and distribution of these variations are largely explained by photon-counting statistics expected for coherent scattering under low-count conditions. Although the present experiment did not provide definitive evidence for intrinsic orientational fluctuations beyond statistical noise, the analysis establishes an important framework for evaluating the detectability of orientational dynamics in time-resolved coherent X-ray scattering experiments and provides practical guidance for future measurements and validation.

## Abstract 2

A statistical framework combining angular X-ray cross-correlation analysis, principal component analysis and photon-counting simulations is presented for evaluating the detectability of orientational fluctuations in time-resolved coherent X-ray scattering experiments.

## 1. Introduction

Understanding orientational fluctuations in anisotropic particle systems is important for clarifying how collective order emerges and destabilizes near phase transitions. In concentrated colloidal systems, such fluctuations are expected to influence not only the formation of liquid-crystalline order but also the stability of mesoscale functionality derived from anisotropic organization. However, directly probing time-dependent orientational order in dense three-dimensional dispersions remains experimentally challenging.

For such systems, coherent X-ray scattering is a powerful approach because it can probe dynamics in optically opaque and concentrated samples. X-ray photon correlation spectroscopy (XPCS) has been widely used to evaluate translational dynamics from temporal fluctuations of coherent speckle patterns over relevant length scales. For anisotropic colloids, XPCS studies have revealed rich concentration-dependent dynamic behavior, including pronounced slowing down of diffusion as liquid-crystalline ordering is approached (Poulos *et al.*, 2010 ▸; Constantin *et al.*, 2010 ▸; Holmqvist *et al.*, 2013 ▸; Hoshino *et al.*, 2019 ▸; Pal *et al.*, 2020 ▸; Hoshino *et al.*, 2023 ▸; Pal *et al.*, 2021 ▸; Kamal *et al.*, 2026 ▸). In parallel, angular X-ray cross-correlation analysis (AXCCA), based on the angular X-ray cross-correlation function (AXCCF), enables orientational information to be extracted from coherent scattering patterns and has been used to characterize local symmetry and hidden structural correlations (Kam, 1977 ▸; Wochner *et al.*, 2009 ▸; Schroer *et al.*, 2015 ▸; Lehmkuhler *et al.*, 2020 ▸; Zaluzhnyy *et al.*, 2019 ▸; Zhao *et al.*, 2024 ▸). AXCCA and fluctuation X-ray scattering have also been applied to detect weak orientational ordering in nanorod solutions and to extract structural information from fluctuation scattering data (Malmerberg *et al.*, 2015 ▸; Pande *et al.*, 2018 ▸; Kurta *et al.*, 2019 ▸).

Anisotropic colloids provide a particularly relevant model system for such studies because their behavior is intrinsically richer than that of spherical particles, with both translational and rotational degrees of freedom contributing to their phase behavior and dynamics. Their static phase behavior depends strongly on particle concentration and aspect ratio, and liquid-crystalline ordering can emerge at high concentrations (Vroege & Lekkerkerker, 1992 ▸; Odriozola, 2012 ▸; Allen, 2019 ▸; Lopes *et al.*, 2021 ▸). In addition, recent advances in sample synthesis and self-assembly have enabled precise control of colloidal liquid-crystalline systems, thereby providing well defined model systems for investigating anisotropic ordering and dynamics (Nakayama & Kato, 2022 ▸; Kato *et al.*, 2021 ▸; van der Kooij & Lekkerkerker, 1998 ▸; Miyamoto & Nakato, 2002 ▸; Xu & Gao, 2011 ▸; Kim *et al.*, 2011 ▸). On the dynamic side, previous theoretical and experimental studies have shown that translational and rotational motions are not necessarily synchronized, and that rotational arrest or heterogeneous rotational dynamics may occur near glassy or liquid-crystalline states (Letz *et al.*, 2000 ▸; Solomon & Spicer, 2010 ▸; Takae & Onuki, 2014 ▸; Segovia-Gutiérrez *et al.*, 2020 ▸; Lama *et al.*, 2024 ▸; Zheng *et al.*, 2011 ▸; Zheng *et al.*, 2014 ▸; Mishra *et al.*, 2013 ▸; Mishra *et al.*, 2014 ▸; Roller *et al.*, 2020 ▸; Roller *et al.*, 2021 ▸; Mishra & Ganapathy, 2015 ▸; Liang *et al.*, 2025 ▸). These findings suggest that orientational order near the isotropic–liquid-crystalline coexistence region may exhibit temporal variability, but the detectability of such fluctuations in three-dimensional concentrated systems has not yet been established.

Despite these advances, temporal fluctuations in orientational order near phase transitions remain insufficiently understood. In particular, when time-resolved coherent scattering is performed at very high frame rates, the photon counts in individual frames can be extremely low. Under such conditions, apparent frame-to-frame variations in angular correlation functions may reflect not only intrinsic sample dynamics but also stochastic fluctuations arising from photon-counting statistics. Distinguishing these contributions is essential if AXCCF is to be used reliably for the analysis of orientational dynamics. A recent study based on time correlations of the AXCCF identified characteristic timescales associated with local rotational symmetry in supercooled colloidal systems (Striker *et al.*, 2023 ▸), but the detectability and interpretation of temporal orientational fluctuations under low-count conditions have not yet been established.

In this study, we investigated concentrated fluorapatite nanorod dispersions near the coexistence region of isotropic and liquid-crystalline phases using time-resolved coherent X-ray scattering at 17.4 kHz. Translational dynamics were evaluated by XPCS, while orientational correlations were examined by AXCCF. To assess the temporal variations observed in the angular correlations, we further combined principal component analysis (PCA) with photon-statistics-based simulations that reproduced the experimentally measured mean intensity distribution under coherent scattering conditions. This combined approach allowed us to examine whether the observed AXCCF fluctuations could be attributed to intrinsic orientational dynamics or were largely explained by counting statistics. The present work therefore provides not only insight into nanorod dynamics near phase coexistence but also a practical framework for assessing the detectability of orientational fluctuations in time-resolved coherent X-ray scattering experiments.

## 2. Methods

### 2.1. Sample preparation

Fluorapatite colloidal nanorods were synthesized based on previous work with modified conditions (Kato *et al.*, 2024 ▸). A 40 m*M* aqueous CaCl_2_ solution containing 0.14 wt% poly­(acrylic acid) (PAA) (*M*_w_ = 1.8 × 10^3^) was mixed with an equal volume of 100 m*M* aqueous Na_2_HPO_4_ and 50 m*M* NaF solution. The mixed solution was stirred for 24 h at 25°C. PAA was purchased from Sigma-Aldrich Japan. CaCl_2_, Na_2_HPO_4_, and NaF were obtained from FUJIFILM Wako Pure Chemical. All reagents were used as received. The resulting colloids were centrifuged, washed with Milli-Q water, and redispersed to adjust the final fluoro­apatite concentration. The size and morphologies of the samples were observed using a field-emission scanning electron microscopy (SEM) (JSM-7800F Prime, operated at 3.0 kV; Jeol) with the samples coated with osmium.

For the X-ray scattering measurements, the samples were sealed in 10 µm-thick quartz capillary tubes with a diameter of 1 mm. To eliminate shear effects that might occur during sample loading into the capillaries, the dispersions were stirred with a thin wire after filling, and then stored vertically along the gravitational direction. To prevent drying, the top ends of the capillaries were sealed with ep­oxy resin. For the isotropic phase, structural differences between short-term and long-term storage were negligible. However, for the liquid-crystalline phase, the behavior may depend on the storage period (Hoshino *et al.*, 2019 ▸). To ensure equilibrium conditions, all X-ray scattering measurements were conducted four months after sealing.

The synthesized colloids were characterized to confirm their suitability as a model system for the present scattering study. SEM analysis showed that the particles were rod-like, with a mean length of 144 ± 26 nm and a mean width of 49 ± 8 nm; the values are presented as mean ± standard deviation for 100 nanorods. Thermogravimetric analysis was used to determine the particle volume fractions of the dispersions, yielding Φ = 0.12, 0.15, 0.18, 0.21, 0.23, 0.24, 0.25, and 0.26. Polarized optical microscopy (POM) confirmed that the samples were in the isotropic phase at Φ = 0.12–0.24, in the nematic phase at Φ = 0.26, and in the coexistence region between the isotropic and nematic phases at Φ = 0.25 (Fig. S1 of the supporting information). These characterizations verify that the sample series appropriately spans the structural transition relevant to the present analysis.

### 2.2. Time resolved partially coherent X-ray scattering measurements

X-ray scattering experiments were conducted at room temperature on beamline BL29XUL at SPring-8, Japan (Tamasaku *et al.*, 2001 ▸). The undulator source and Si(111) monochromator were tuned to 12.40 keV, and higher-harmonic X-rays were removed using Rh-coated mirrors. The sample was irradiated with partially coherent X-rays obtained by passing the beam through 20 µm × 20 µm slits. The scattered X-rays were detected using an integrating-type high-speed detector, CITIUS, with a pixel size of 72.6 µm. 30000 time-resolved scattering images were acquired at 17.4 kHz with a total exposure time of approximately 1.7 s. The detector was operated with a gain setting corresponding to 92 digital numbers (DN) per photon. The detector was mounted approximately 6 m downstream from the sample. For each concentration sample, measurements were taken at a position approximately 1 cm from the bottom of the capillary tube at 11 locations at 0.1 mm intervals, each with a range of 1 mm. The lowest measurement point was defined as *Z* = 0 mm, and the highest measurement point was *Z* = 1 mm.

Because the photon counts per pixel per frame were limited under the present high-speed measurement conditions, direct evaluation of the orientational order parameter from single-frame azimuthal profiles was not feasible. Therefore, frame-wise AXCCF analysis combined with PCA was adopted as a statistical approach to evaluate temporal variations in orientational anisotropy.

## 3. Results and discussion

### 3.1. Static structure characterization based on SAXS

**Figure 1:**
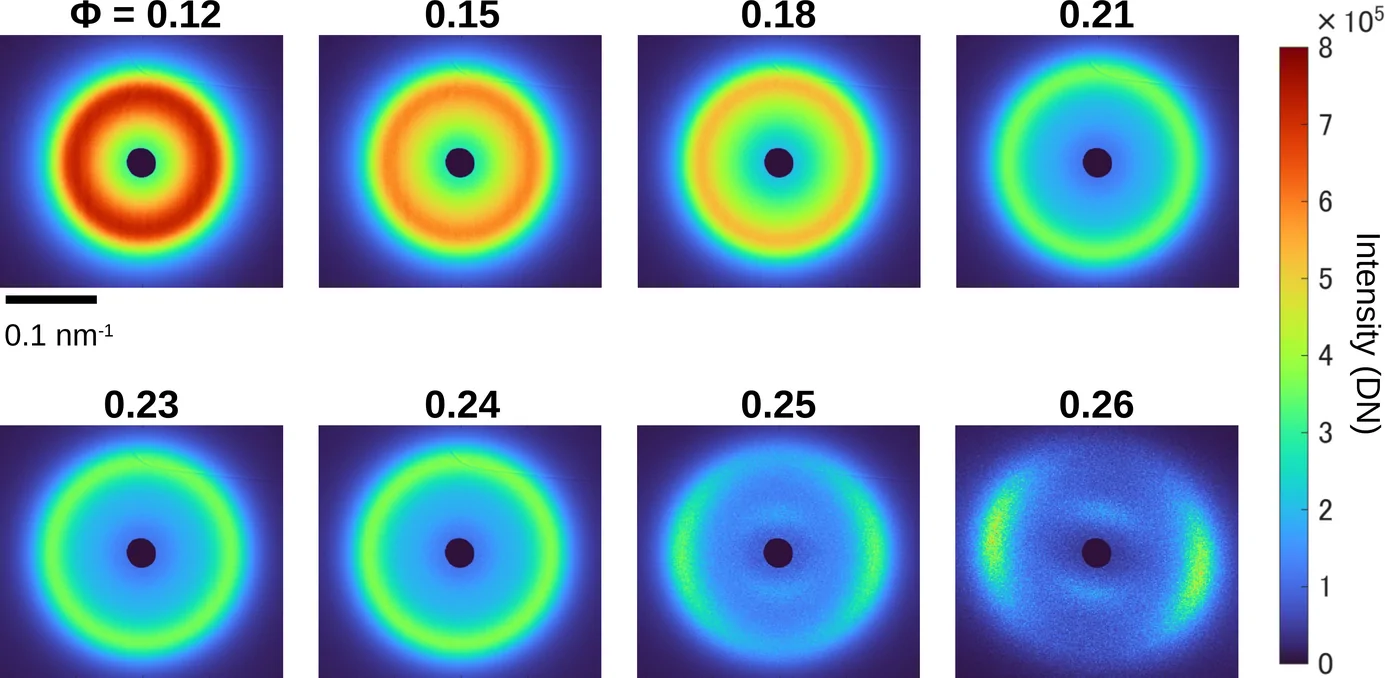
Integrated scattering images for the various concentrations.

**Figure 2:**
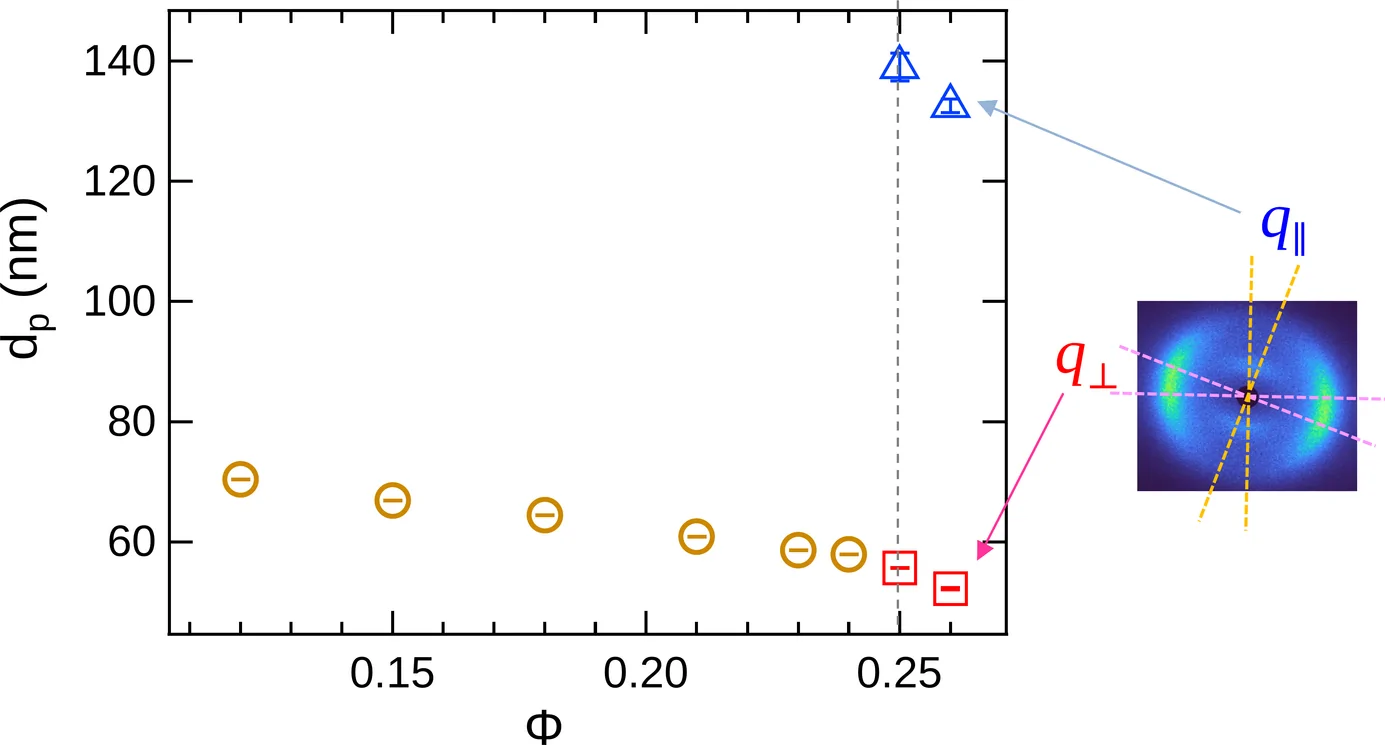
Concentration dependence of the characteristic interparticle distance *d*_p_. For Φ ≤ 0.24, *d*_p_ is plotted as circles. For Φ ≥ 0.25, *d*_p_ for the long-axis (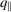) and the short-axis (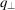) directions is plotted as triangles and squares, respectively.

Fig. 1 ▸ shows the integrated scattering images obtained at the central measurement position (*Z* = 0.5) for various concentrations. In the isotropic phase (Φ = 0.12–0.24), isotropic scattering patterns were observed, whereas anisotropic scattering patterns appeared in the coexistence and nematic phases (Φ = 0.25–0.26). The global director orientation was most likely influenced by weak effects from the capillary walls or gravity. Fig. 2 ▸ presents the concentration dependence of the characteristic interparticle distance *d*_p_ = 2π/*q*_p_, estimated from the peak position *q*_p_ of the effective structure factor *S*(*q*), which was obtained by dividing each scattering profile by that of a dilute dispersion (Φ = 0.005). Here, *d*_p_ should be regarded as a characteristic length scale associated with the dominant interparticle correlations rather than a direct geometrical particle-to-particle distance, because *q*_p_ can be influenced by particle packing and interparticle correlations. As shown in Fig. S2, the scattering profile of the dilute dispersion was well described by that of an ellipsoidal dispersion (Hashimoto, 2022 ▸). The plotted values represent the averages over 11 measurement points for each concentration. In the isotropic phase (Φ = 0.12–0.24), *d*_p_ decreased with increasing concentration, whereas, at Φ = 0.25 and 0.26, the characteristic interparticle distances were separately analyzed along the short (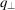) and long axes (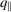) by extracting data within a range of ±10°, as illustrated on the right-hand side of Fig. 2 ▸. As a representative example of the structure factor, Fig. S3 shows 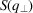 at Φ = 0.25, where the *Z* dependence of the peak position was negligibly small.

**Figure 3:**
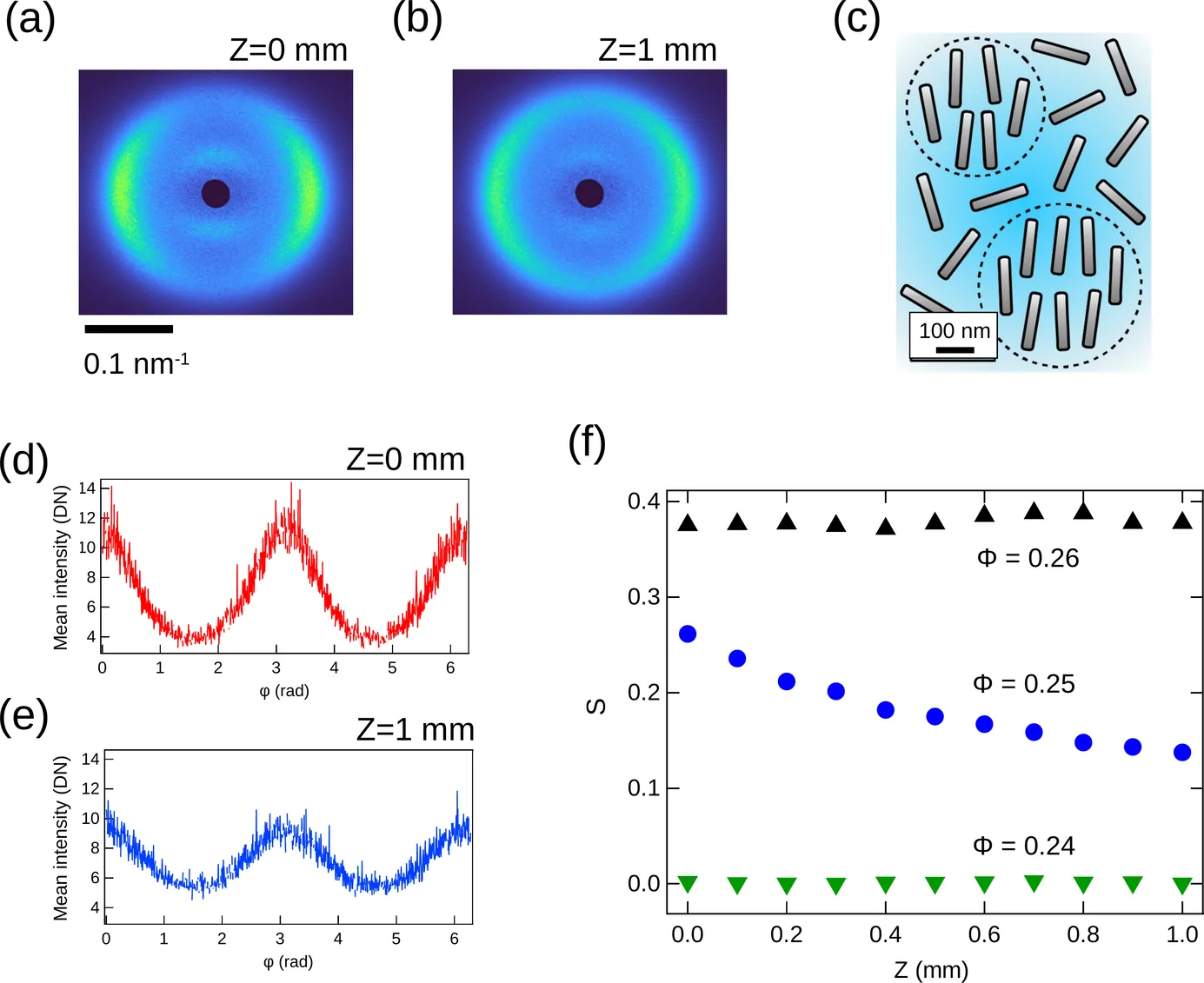
Panels (*a*) and (*b*) show integrated scattering images for *Z* = 0 and 1 mm. (*c*) Schematic of the isotropic/liquid-crystalline phase coexistence structure. Panels (*d*) and (*e*) show the frame-averaged azimuthal profile obtained at the high-*q* peak position for *Z* = 0 and 1 mm. (*f*) *Z*-dependence of the orientational order parameter *S* for Φ = 0.24, 0.25, and 0.26.

Here, we focus on the scattering patterns observed in the coexistence phase (Φ = 0.25). Figs. 3 ▸(*a*) and 3 ▸(*b*) depict integrated scattering images at *Z* = 0 mm and 1 mm, respectively, for the isotropic/liquid-crystalline coexisting phase (Φ = 0.25). In this phase, liquid-crystalline and isotropic regions coexist, as schematically illustrated in Fig. 3 ▸(*c*). The schematic illustration is based on our previous POM observations near the coexistence region in a similar nanorod colloidal system (Nakayama *et al.*, 2018 ▸). According to those results, in the coexistence phase, the fraction of liquid-crystalline domains changes significantly with only a slight variation in concentration. It should be noted that the schematics are intended to qualitatively highlight such local heterogeneity and temporal fluctuations, while the overall nematic alignment is maintained across the macroscopic scattering volume, as shown by the anisotropy in the scattering images. At *Z* = 0 mm, the scattering image is clearly anisotropic, reflecting the structure of the oriented nanorods, as depicted in Fig. 3 ▸(*c*). The orientational order parameter *S* can be calculated using the azimuthal ϕ profile *I*(*q*, ϕ) obtained at the high-*q* peak position *q* = 0.113 nm^−1^ of the structure factor. Based on the average of the second Legendre polynomial *P*_2_, *S* is calculated using the following equation,
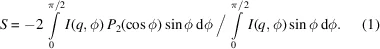
From this calculation, *S* = 0.26 is obtained at *Z* = 0 mm. In contrast, the anisotropy of the scattering image at *Z* = 1 mm [Fig. 3 ▸(*b*)] was reduced, with *S* = 0.14. The same analysis of the scattering images from *Z* = 0 to 1 mm was performed to obtain the *Z* dependence of *S*, as shown in Fig. 3 ▸(*f*). This indicates that, in the coexisting phases, a slight density gradient (*a*) exists in the sample cell, which in turn leads to a variation in the orientational order. For comparison, the values of *S* at Φ = 0.24 and 0.26 are also plotted in Fig. 3 ▸(*f*). At Φ = 0.24, corresponding to the isotropic phase, *S* ≃ 0, whereas, at Φ = 0.26, *S* was approximately 0.38 with little *Z* dependence.

### 3.2. Translational motion analysis based on XPCS

**Figure 4:**
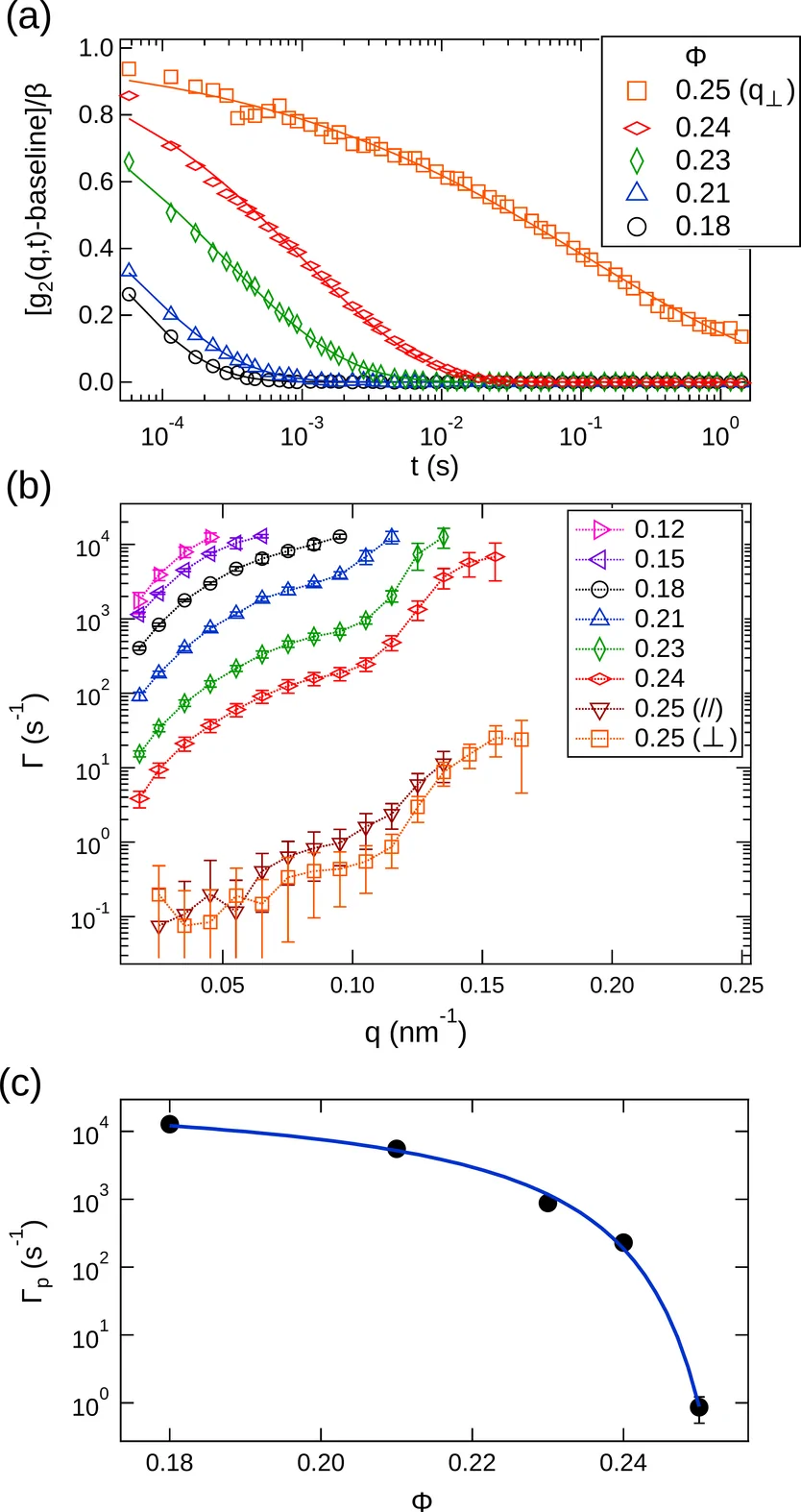
(*a*) *g*_2_ at *q*_p_ for various concentrations. The solid lines represent the fitted curves obtained using equation (3). At Φ = 0.25, *g*_2_ obtained in the direction of the short axis (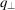). (*b*) *q* dependence of Γ for various Φ. (*c*) Concentration dependence of Γ_p_. The solid line is the fitting curve for Γ_p_ = *C*exp[*F*Φ/(Φ_0_ − Φ)].

From the time-resolved scattering data, the following time autocorrelation functions for the scattering intensity *I*(**q**, *t*) at scattering wavevector **q** and time *t* were calculated,
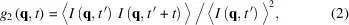
where the angular brackets indicate time averaging. Fig. 4 ▸(*a*) shows the obtained *g*_2_ at *q*_p_, which is the peak position of the structure factor, for various concentrations at *Z* = 0.5 mm. At Φ = 0.25, *g*_2_ obtained in the direction of the short axis (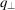) within a range of ±10° is shown. All the measured *g*_2_ values were well expressed by the following equation,
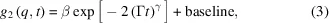
where β is the speckle contrast, and Γ and γ are the relaxation rate and the stretched or compressed exponent, respectively. The speckle contrast under the present experimental conditions was independently estimated from *g*_2_ measured for a more concentrated nanorod dispersion (Φ = 0.31) 11 days after sealing, using the same experimental configuration. Because the relaxation of this sample was sufficiently slow, β ≃ 0.06 was estimated from the short-time amplitude of *g*_2_ − 1 (Fig. S4). The *q* dependence of Γ for various Φ is shown in Fig. 4 ▸(*b*), where the data at Φ = 0.25 were obtained from the long-axis direction 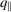 and the short-axis direction 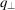 within a range of ±10°. At Φ = 0.26, no significant relaxations were observed, probably because it was slower than the accessible time window of the measurement. For all *q*, Γ decreases with increasing concentration. In addition, since γ < 1 (see Fig. S5) and Γ ∝ *q*^*n*^ with *n* > 2 (Fig. S6), the dynamical behavior can be regarded as subdiffusive (Sprakel *et al.*, 2007 ▸; Guo *et al.*, 2012 ▸; Hoshino *et al.*, 2013 ▸; Frey *et al.*, 2025 ▸). In concentrated colloidal systems, the collective relaxation rate can be influenced by both the static structure factor and hydro­dynamic interactions. In the present anisotropic nanorod system, these effects may additionally depend on the direction of motion relative to the particle orientation. Thus, the *q* dependence of Γ observed in Fig. 4 ▸(*b*) may reflect contributions from de Gennes narrowing and hydro­dynamic interactions. At Φ = 0.25, 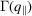 tends to be larger than 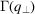, which is consistent with the previous reports (Hoshino *et al.*, 2019 ▸; Pal *et al.*, 2020 ▸; Pal *et al.*, 2022 ▸). This anisotropy indicates that the translational dynamics in the coexisting phase depend on the direction relative to the nanorod orientation.

The concentration dependence of Γ_p_ (Γ at *q*_p_), averaged over 11 locations at *Z* = 0–1 mm, is shown in Fig. 4 ▸(*c*). Γ_p_ exhibits rapid slowing behavior as the concentration approaches the coexisting phase from the isotropic phase. It follows the relationship Γ_p_ = 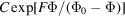 where *C* and *F* are constants, and Φ_0_ = 0.26 was obtained. This behavior is consistent with the findings reported by Pal *et al.* (2021 ▸). Furthermore, Γ_p_ tends to increase slightly with increasing *Z* (shown in Fig. S7). This increase is attributed to the effect of density gradients.

### 3.3. Analysis of orientational fluctuations using AXCCF and PCA

**Figure 5:**
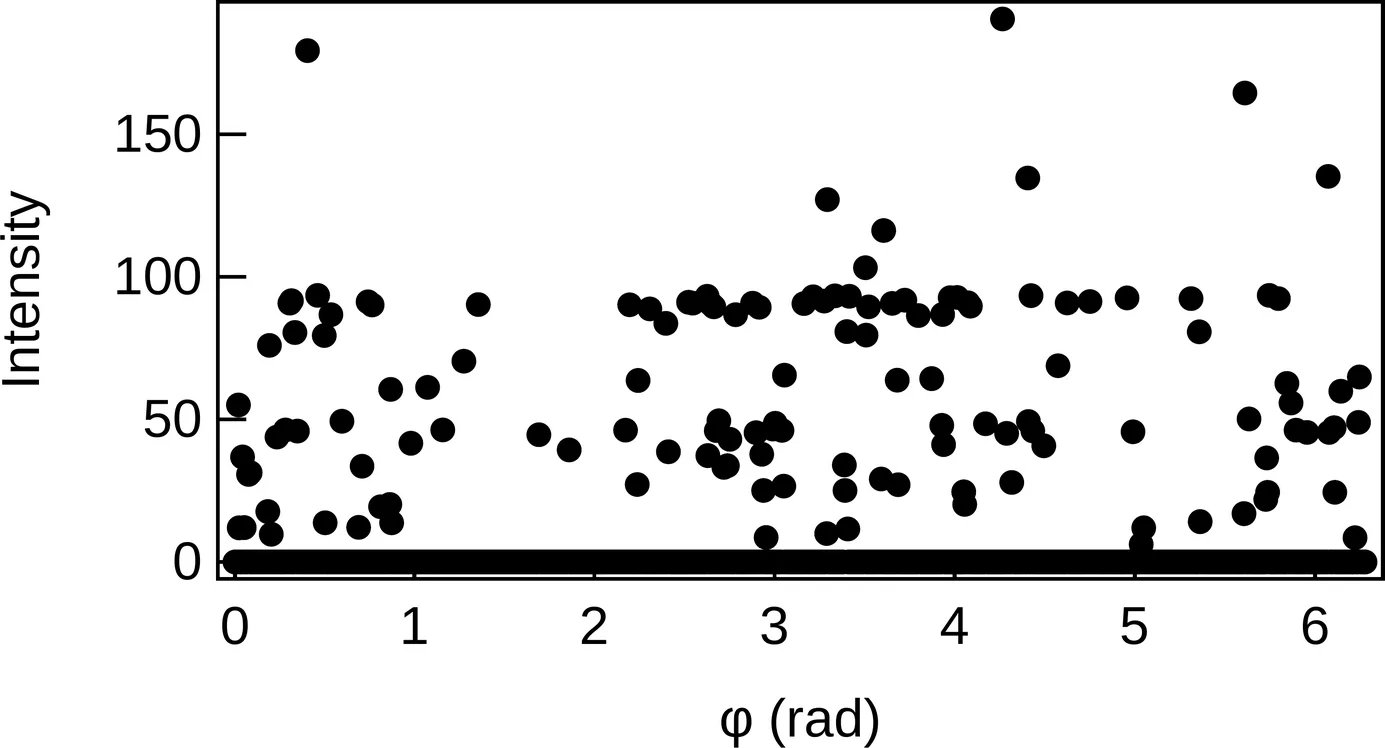
Representative azimuthal profile extracted from a single-frame scattering image at *q* = *q*_p_ with a one-pixel width.

**Figure 6:**
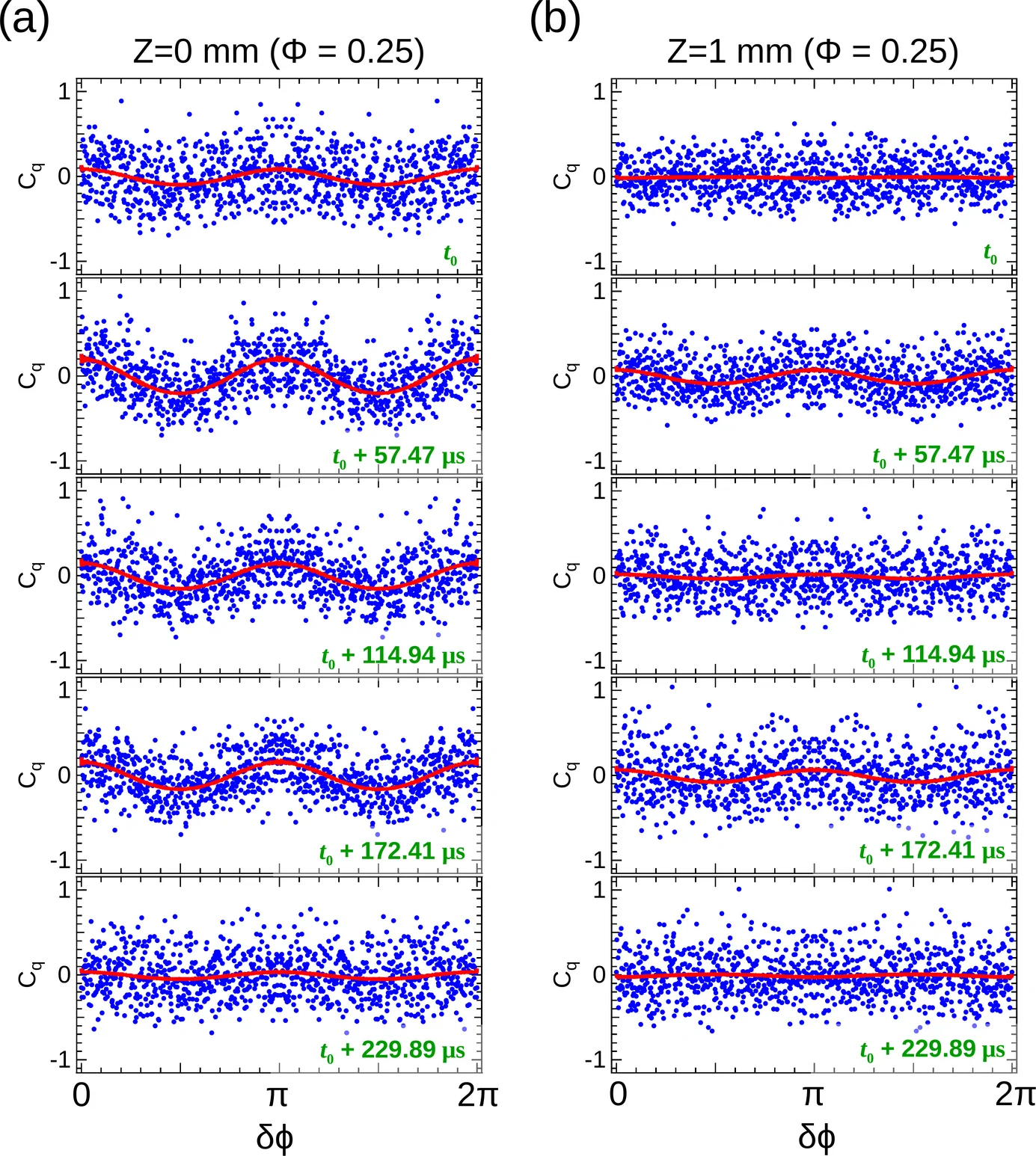
AXCCF calculated for the azimuthal profile at *q* = 0.1130 nm^−1^ from five consecutive images (17.4 kHz) at *Z* = 0 mm (*a*) and *Z* = 1 mm (*b*) for Φ = 0.25. Red lines: the first principal component obtained from PCA of the experimental AXCCFs.

As shown above, there is diversity in the orientational order and translational dynamics in the coexisting phases. We therefore examined whether time-resolved coherent scattering could provide information on temporal variations in the orientational correlations. In Fig. 3 ▸(*d*) and 3(*e*), the average orientational order was discussed based on the 1.7 s integrated images. Ideally, temporal fluctuations of the orientational order could also be assessed by analyzing time-resolved scattering images in detail. However, because the scattering intensity in each individual frame was too weak, it was not possible to obtain a physically meaningful orientational order parameter. A representative azimuthal profile extracted from a single-frame scattering image at *q* = *q*_p_ with a one-pixel width is shown in Fig. 5 ▸. Considering that one detected photon corresponds to an intensity of 92 DN in the present detector system, it is evident that most pixels in the azimuthal profiles contain no more than two photon counts per frame. Thus, for each scattering image obtained at 17.4 kHz, AXCCF was calculated for the azimuthal profile at *q* = 0.113 nm^−1^ by the following equation,
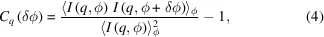
where 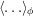 indicates angular averaging. The speckle size estimated under the present experimental conditions was approximately 20–30 µm, which is smaller than the detector pixel size of 72.6 µm. Thus, the speckle pattern was undersampled, and spatial averaging within individual detector pixels is expected to reduce the speckle contrast and the sensitivity to coherent intensity fluctuations. Nevertheless, a finite speckle contrast (∼0.06) was observed experimentally, and the following analysis evaluates the angular variations in the detector-sampled intensity distribution. Following the discrete-grid formulation of the AXCCF described by Lapkin *et al.* (2022 ▸), the correlation was evaluated directly from pairs of detector pixels without first averaging the intensities into azimuthal bins. Fig. 6 ▸ shows the obtained *C*_*q*_ from five consecutive images at *Z* = 0 and 1 mm as representative data. At *Z* = 0 mm, periodic correlations were clearly observed in the second to fourth frames. At *Z* = 1 mm, some frames, such as the second and fourth, exhibit periodic behavior, whereas the others are almost flat.

These results show pronounced frame-to-frame variations in the periodic features of the individual AXCCFs. However, such variations cannot be directly attributed to fluctuations in orientational order, because an individual AXCCF may contain contributions from both orientational correlations and the instantaneous translational arrangement of the particles. In the present measurements, the apparent AXCCF variations occur on a frame-to-frame timescale, which is substantially shorter than the translational relaxation observed by XPCS. This difference in timescale alone, however, does not establish an orientational origin, particularly under the present low-photon-count conditions. Because the individual *C*_*q*_ profiles are highly noisy, we examine below whether the observed variations can be explained by photon-counting statistics before considering their physical origin. The influence of experimental noise on X-ray cross-correlation and fluctuation scattering analyses has also been recognized in previous studies (Lehmkühler *et al.*, 2014 ▸; Malmerberg *et al.*, 2015 ▸). To evaluate the periodic features in *C*_*q*_ in a quantitative and unbiased manner, we employed PCA. This approach is advantageous because the data exhibit relatively simple periodic patterns, while the large number of frames and the noise level make manual inspection or simple smoothing procedures impractical. PCA has also been successfully used to extract essential information from noisy datasets (Matos *et al.*, 2007 ▸; Kusaka *et al.*, 2019 ▸; Guccione *et al.*, 2020 ▸).

In PCA, the first principal component is determined to maximize the variance, and the subsequent principal components are orthogonal to the previously determined components. As a result, standardized *C*_*q*_(δϕ), 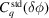, can be expressed as a linear sum over orthogonal bases as follows,
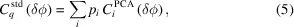
where 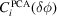 is the *i*th principal component and *p*_*i*_ is its weighting coefficient. The principal components form a basis obtained without preconditions, with the first principal component (*i* = 1) containing the most variable information characterizing the data.

**Figure 7:**
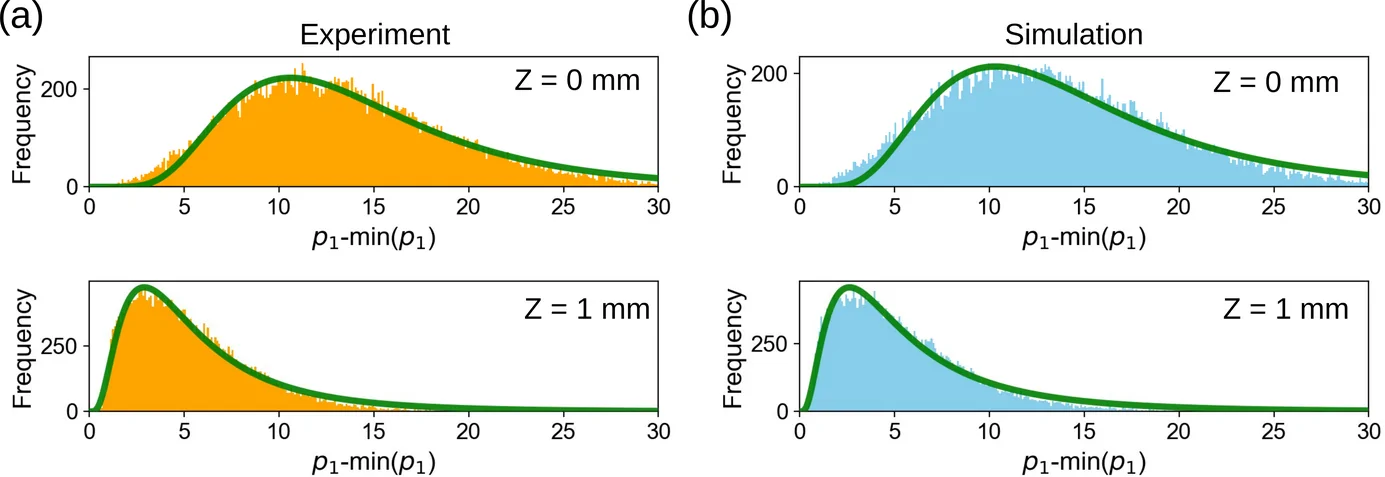
Distributions of the PC1 scores at *Z* = 0 and 1 mm for experimental data (*a*) and simulated data (*b*). The simulated data were projected onto the PCA basis obtained from the experimental AXCCFs. Green lines: fitting curves using the lognormal distribution.

PCA was performed using the experimental *C*_*q*_(*q* = *q*_*p*_) data for Φ = 0.25, comprising 330000 frames collected at 11 positions from *Z* = 0 to 1 mm (30000 valid frames at each position). Before PCA, each angular component was standardized by mean-centering and scaling to unit variance using the mean and standard deviation determined from the experimental dataset. The PCA basis was then determined solely from these standardized experimental AXCCFs. As shown in Fig. 6 ▸, the first principal component exhibits twofold symmetry with a period of π, originating from the orientational order of the peaks associated with the inter-particle distances along the short axis direction. It is evident from Fig. 6 ▸ that the magnitude of the first principal component, *p*_1_, fluctuates over time and varies significantly with the *Z* position. The distributions of *p*_1_ at *Z* = 0 and 1 mm for Φ = 0.25 are shown as histograms in Fig. 7 ▸, and all the distributions at the 11 positions between *Z* = 0 and 1 mm are shown in Fig. S8(*a*).

As an additional cross-check, we compared the AXCCF averaged over all individual frames with the AXCCF calculated directly from the time-averaged scattering pattern. At both *Z* = 0 and 1 mm, the angular dependences of the two AXCCFs were in close agreement (Fig. S9).

**Figure 8:**
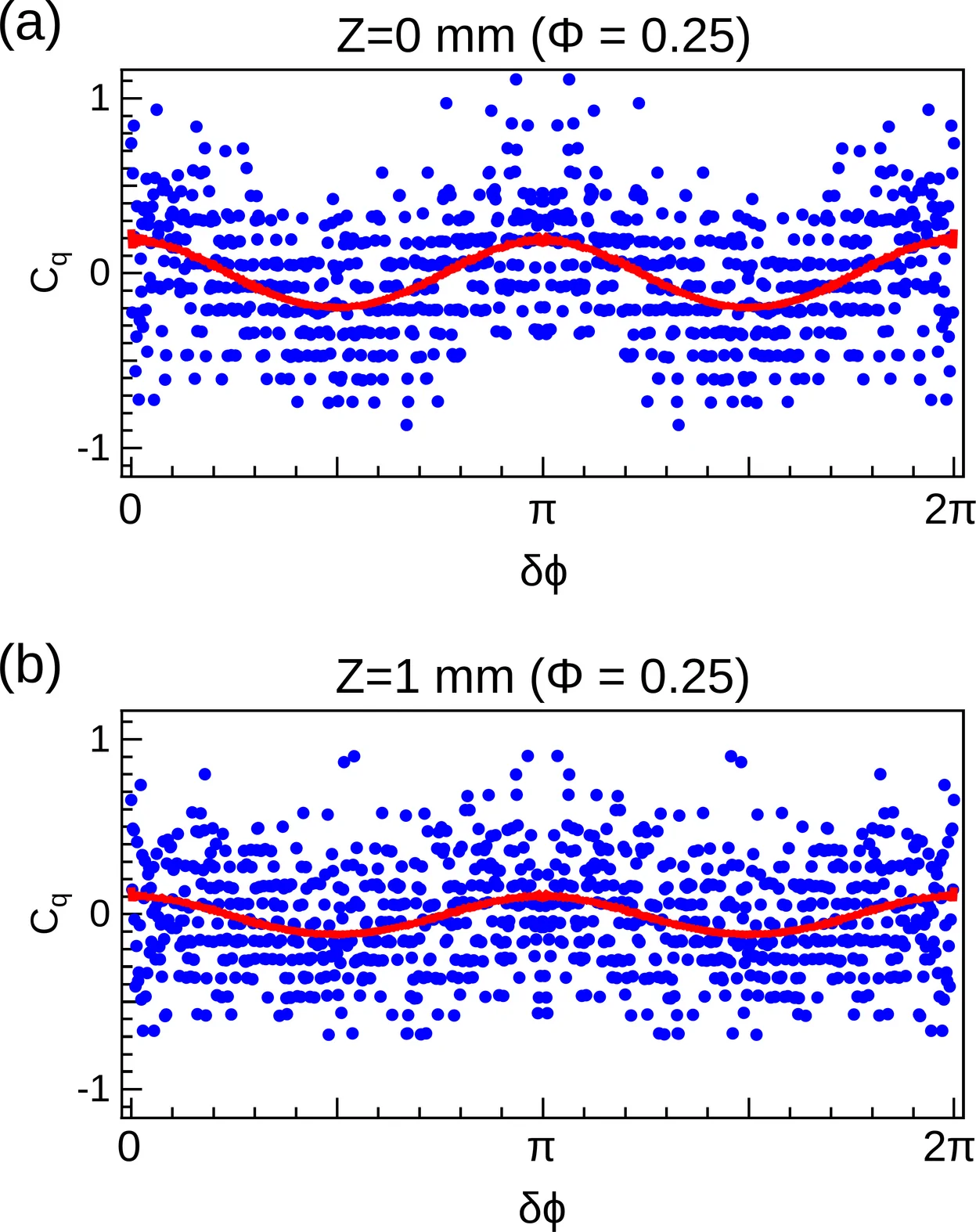
Representative AXCCF calculated for simulated data at *Z* = 0 mm (*a*) and *Z* = 1 mm (*b*). Red lines: the first principal component obtained from PCA of the experimental AXCCFs.

We next examined the origin of the temporal fluctuations in the AXCCF observed in Fig. 6 ▸ and of the distributions shown in Fig. 7 ▸(*a*) from the viewpoint of photon-counting statistics. For highly coherent scattering, the probability density of the instantaneous intensity is described by a gamma distribution. Photon detection at a given integrated intensity follows Poisson statistics. By combining these two distributions, the probability of detecting *K* photons is given by a negative binomial distribution as follows,
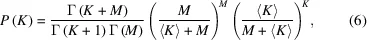
where *M* = 1/β is the effective number of coherent modes and *K* is the mean photon count. Based on this probability distribution, we generated 30000 azimuthal profiles using the experimentally determined speckle contrast of β = 0.06, corresponding to *M* = 1/β. For each detector pixel, the mean photon count *K*_*i*_ was determined from the experimentally measured mean intensity over all frames by assuming 92 DN per photon. Thus, the simulations reproduced both the experimentally measured mean azimuthal intensity distribution and the absolute photon-count level at each *Z* position. As shown in Fig. S10, the average azimuthal profile calculated from the simulated data agrees well with the experimental profile, confirming that the simulated dataset reproduces the mean experimental scattering anisotropy. AXCCF was then applied to the 30000 simulated azimuthal profiles using equation (4), in the same manner as for the experimental data. Representative examples are shown in Fig. 8 ▸. In the simulation, AXCCF takes discrete values because the analysis is based on discrete photon counts, and under the present low-count conditions. Nevertheless, the simulated data exhibit features closely resembling those of the experiment: some frames show clear periodicity, whereas others do not.

The simulated AXCCFs were standardized using the same mean and standard deviation determined from the experimental dataset and then projected onto the fixed PCA basis obtained solely from the experimental AXCCFs. The resulting PC1-score distributions at *Z* = 0 and 1 mm are shown in Fig. 7 ▸(*b*), while those for all positions are presented in Fig. S8(*b*). These histograms closely resemble those obtained from the experimental data. Specifically, at *Z* = 0 mm, the distribution peaks at a relatively large value and is comparatively symmetric, whereas, at *Z* = 1 mm, the peak shifts to smaller values and the distribution exhibits a long tail toward larger values.

**Figure 9:**
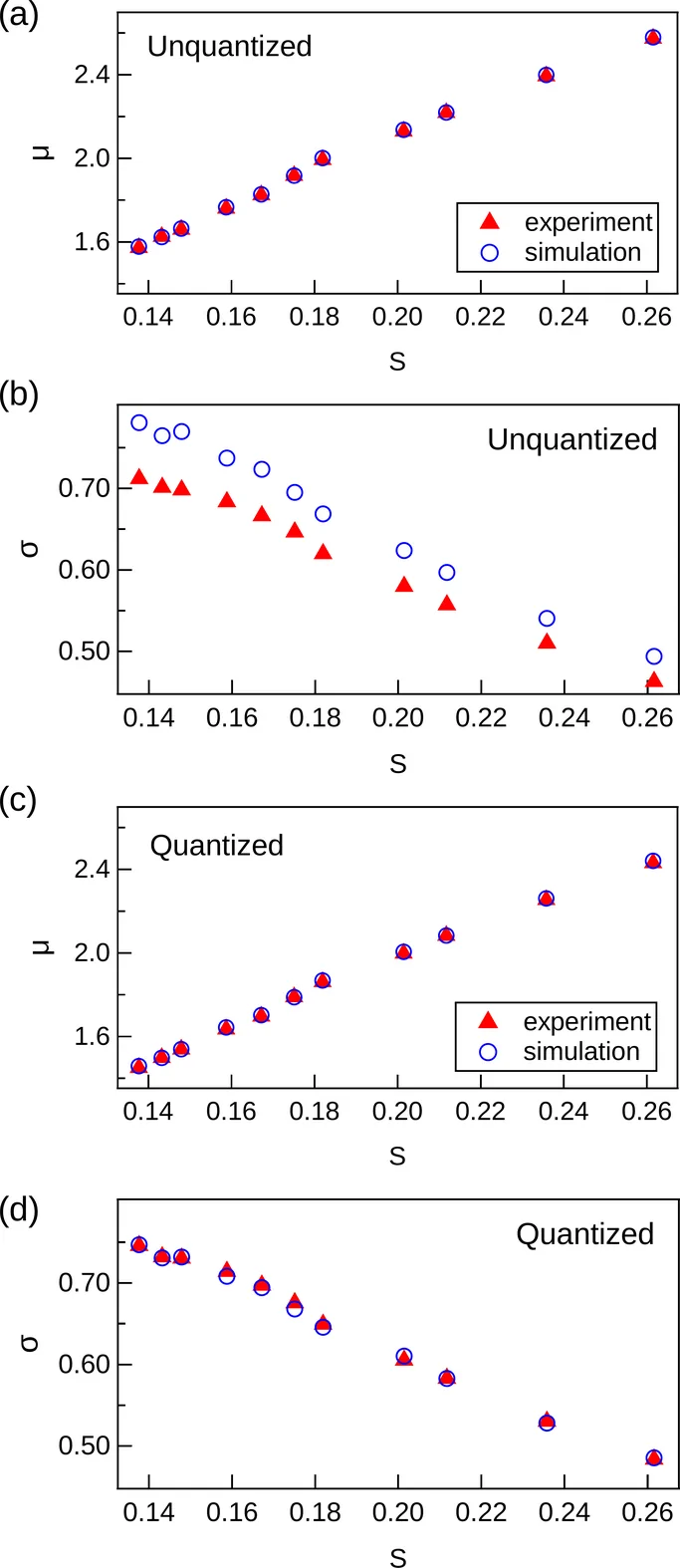
*S* dependence of the lognormal-distribution parameters μ and σ obtained by comparing the simulated data with the unquantized experimental data [(*a*) μ; (*b*) σ] and with the quantized experimental data [(*c*) μ; (*d*) σ].

For quantitative comparison, the histograms were fitted using the following lognormal distribution function (Crow & Shimizu, 1988 ▸),
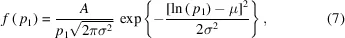
where *A* is the coefficient, μ is the peak position, and σ is a variable related to the shape of the distribution. Figs. 9 ▸(*a*) and 9 ▸(*b*) show the *S* dependences of μ and σ, respectively, obtained from the unquantized experimental data and the simulated data. The experimental and simulated results show similar *S* dependences, with particularly good agreement in μ. In contrast, a systematic difference is observed in σ, with the simulated distributions being slightly broader than the experimental distributions.

Because the experimental detector signals were recorded as continuous-valued DN data, whereas the simulation was based on integer photon counts, we examined whether this difference in signal representation contributed to the discrepancy. We therefore repeated the AXCCF and PCA analyses after quantizing the experimental detector signals into integer photon counts. The detector output at each pixel was divided by 92 DN photon^−1^ and then assigned to non-negative integer photon counts using thresholds of 0.5, 1.5, 2.5,… photons. Representative single-frame azimuthal profiles before and after quantization are shown in Fig. S11.

The PC1-score distributions at all 11 positions are shown in Fig. S12. After quantization, the systematic *Z* dependence of the distributions was retained, and the experimental distributions showed good agreement with the corresponding simulated distributions. Figs. 9 ▸(*c*) and 9(*d*) show the *S* dependences of μ and σ, respectively, obtained from the quantized experimental data and the simulated data. After quantization, the agreement between the experimental and simulated results improved not only for μ but also for σ. This result indicates that the discrepancy observed between the unquantized experimental data and the simulation can be largely attributed to the difference in signal representation between the continuous-valued experimental detector output and the integer photon counts used in the simulation. Furthermore, the good agreement between the quantized experimental data and the simulation based on photon-counting statistics supports our conclusion that most of the frame-to-frame AXCCF variations observed under the present low-photon-count conditions can be explained by photon-counting statistics.

These results indicate that most of the frame-to-frame variations in the AXCCFs shown in Fig. 6 ▸ can be explained by photon-counting statistics under the present low-photon-count conditions. This finding highlights the need for caution when interpreting temporal variations in AXCCFs obtained in high-speed coherent scattering experiments with limited photon statistics.

**Figure 10:**
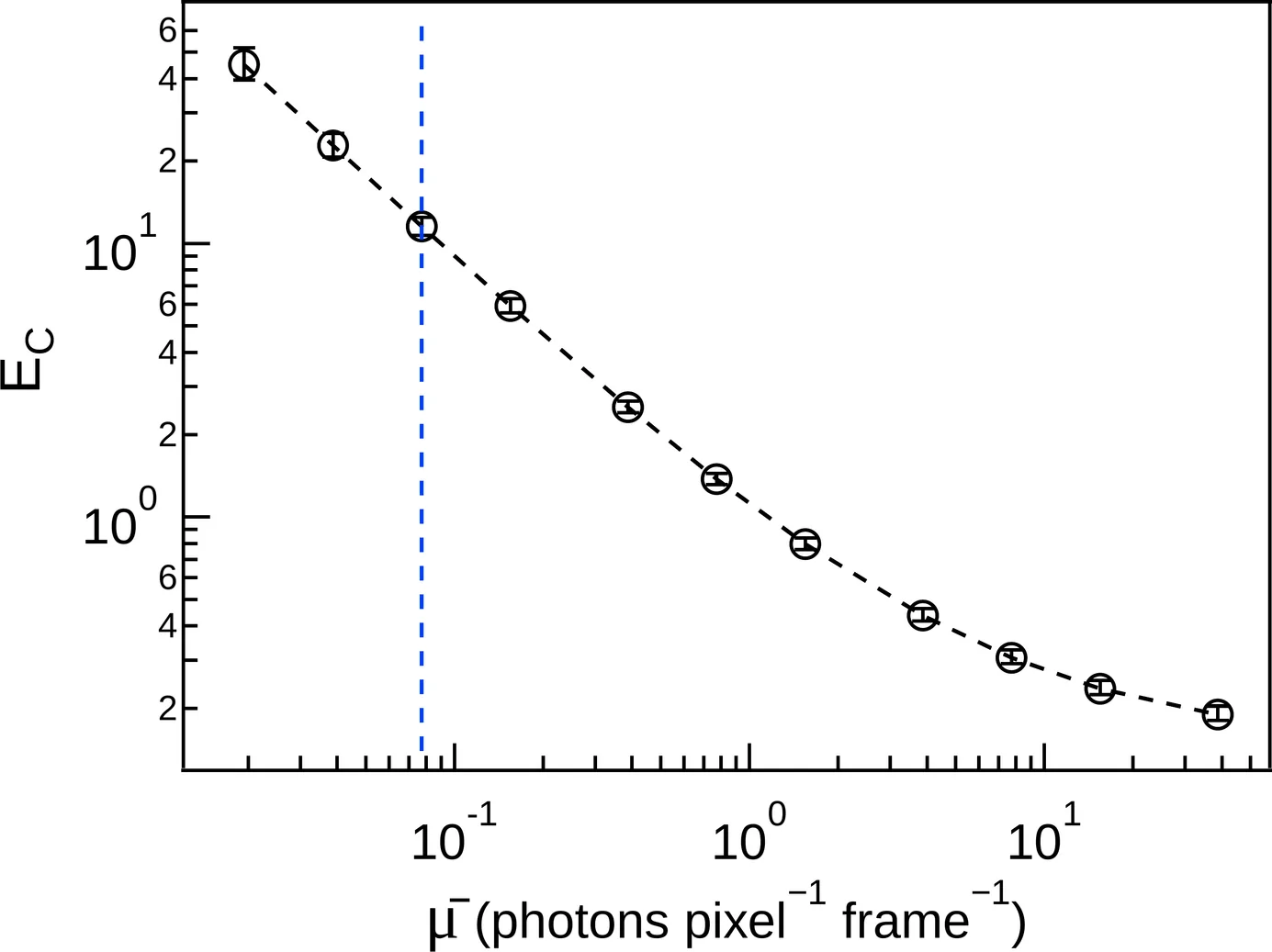
Photon-count dependence of the normalized discrepancy *E*_*C*_ between the simulated and reference AXCCFs. The simulations were performed with a speckle contrast of β = 0.06 while varying the mean photon count per pixel per frame. The symbols and error bars represent the median and interquartile range, respectively. The blue dashed line indicates the mean photon count in the present experiment (0.077 photons pixel^−1^ frame^−1^).

Furthermore, to estimate the photon-count requirements for future experiments, we performed simulations while varying the mean photon count. As the reference azimuthal intensity distribution, we used a profile reconstructed from the experimental azimuthal intensity averaged over all frames at *Z* = 1 mm using the *n* = 0, 2, and 4 Fourier components (Fig. S13). The shape of this profile was fixed, and scattering intensities were generated with a fixed speckle contrast while varying only the mean photon count per pixel per frame. To quantify the AXCCF variations arising from photon statistics, we evaluated the normalized discrepancy,
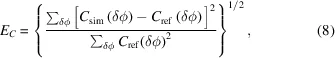
between the simulated AXCCFs and the reference AXCCF calculated from the reference profile. Fig. 10 ▸ shows the dependence of *E*_*C*_ on the mean photon count. The mean photon count under the present experimental conditions was approximately 0.077 photons pixel^−1^ frame^−1^, for which *E*_*C*_ was relatively large. With increasing photon count, *E*_*C*_ decreased rapidly, falling below 1 at approximately 1.5 photons pixel^−1^ frame^−1^ and reaching approximately 0.5 at 4 photons pixel^−1^ frame^−1^.

These results suggest that, for more reliable single-frame evaluation of weak azimuthal anisotropy comparable with that observed in the present experiment, a mean photon count of at least approximately 1 photon pixel^−1^ frame^−1^ may serve as a practical guideline, while several photons pixel^−1^ frame^−1^ would be desirable to substantially reduce the influence of statistical fluctuations. These values depend on the azimuthal intensity distribution, speckle contrast, and analysis conditions used in the present study and therefore should not be regarded as universal thresholds. Improved photon statistics could be achieved through higher coherent flux, frame accumulation within a time window that preserves the relevant dynamics, or optimized *q*-space binning.

To further assess the robustness and temporal characteristics of the analysis, we performed several complementary analyses. The temporal correlation of the AXCCF showed no significant relaxation (Fig. S14). Fourier analysis confirmed that the second-order component was dominant and strongly correlated with the PC1 score (Figs. S15 and S16). In addition, the statistical variation in the orientational order parameter *S* decreased with increasing frame accumulation, although this improvement was accompanied by a loss of temporal resolution (Fig. S17). These results support the interpretation of the AXCCF-PCA analysis and clarify the trade-off between statistical precision and temporal resolution.

## 4. Conclusions

We investigated the dynamics of concentrated fluorapatite nanorod dispersions near the coexistence region of isotropic and liquid-crystalline phases using time-resolved partially coherent X-ray scattering at 17.4 kHz. XPCS analysis revealed a marked slowing down of translational dynamics as the concentration approached the coexistence phase from the isotropic side, consistent with previous observations for anisotropic colloidal systems.

To explore possible temporal fluctuations in orientational order, AXCCF analysis was applied to the time-resolved scattering images, and the resulting frame-by-frame variations were further evaluated by PCA. Although temporal variations in the AXCCF were observed and the PCA-derived distributions depended systematically on the orientational order parameter *S*, comparison with statistically simulated datasets demonstrated that the observed variations can be largely reproduced by photon-counting statistics under the present low-count conditions.

These results show that caution is required when interpreting temporal fluctuations in AXCCF obtained from ultrafast coherent scattering experiments with limited photon statistics. At the same time, the present study establishes a practical framework for evaluating the detectability of orientational fluctuations by combining AXCCF, PCA, and photon-statistics-based simulation. This approach should be useful for designing future experiments aimed at identifying intrinsic orientational dynamics beyond statistical noise in anisotropic soft-matter systems.

## Supplementary Material

Figures S1 to S17. DOI: 10.1107/S160057752600980X/mad5010sup1.pdf

## References

[bb1] Allen, M. P. (2019). *Mol. Phys.* **117**, 2391–2417.

[bb3] Constantin, D., Davidson, P., Freyssingeas, E. & Madsen, A. (2010). *J. Chem. Phys.* **133**, 224902. 10.1063/1.3509399 21171697

[bb4] Crow, E. L. & Shimizu, K. (1988). *Lognormal Distributions: Theory and Applications.* New York: Dekker.

[bb6] Frey, M., Neuber, N., Riegler, S. S., Cornet, A., Chushkin, Y., Zontone, F., Ruschel, L. M., Adam, B., Nabahat, M., Yang, F., Shen, J., Westermeier, F., Sprung, M., Cangialosi, D., Di Lisio, V., Gallino, I., Busch, R., Ruta, B. & Pineda, E. (2025). *Nat. Commun.* **16**, 4429. 10.1038/s41467-025-59767-2 PMC12075684 40360505

[bb7] Guccione, P., Lopresti, M., Milanesio, M. & Caliandro, R. (2020). *Crystals* **11**, 12.

[bb8] Guo, H., Bourret, G., Lennox, R. B., Sutton, M., Harden, J. L. & Leheny, R. L. (2012). *Phys. Rev. Lett.* **109**, 055901. 10.1103/PhysRevLett.109.055901 23006188

[bb9] Hashimoto, T. (2022). *Principles and Applications of X-ray, Light and Neutron Scattering.* Singapore: Springer Nature.

[bb11] Holmqvist, P., Meester, V., Westermeier, F. & Kleshchanok, D. (2013). *J. Chem. Phys.* **139**, 084905. 10.1063/1.4818532 24007035

[bb12] Hoshino, T., Murakami, D., Tanaka, Y., Takata, M., Jinnai, H. & Takahara, A. (2013). *Phys. Rev. E* **88**, 032602. 10.1103/PhysRevE.88.032602 24125287

[bb13] Hoshino, T., Nakayama, M., Fujinami, S., Nakatani, T., Kohmura, Y. & Kato, T. (2019). *Soft Matter* **15**, 3315–3322. 10.1039/c9sm00101h 30810584

[bb14] Hoshino, T., Nakayama, M., Hosokawa, Y., Mochizuki, K., Kajiyama, S., Kohmura, Y. & Kato, T. (2023). *Nanoscale Adv.* **5**, 3646–3654. 10.1039/d3na00183k PMC10334381 37441264

[bb16] Kam, Z. (1977). *Macromolecules* **10**, 927–934.

[bb17] Kamal, M. A., Zinn, T. & Pal, A. (2026). *Phys. Rev. Res.* **8**, 013341.

[bb18] Kato, R., Mikami, T. & Kato, T. (2024). *Adv. Mater.* **36**, 2404396. 10.1002/adma.202404396 38877780

[bb19] Kato, T., Gupta, M., Yamaguchi, D., Gan, K. P. & Nakayama, M. (2021). *Bull. Chem. Soc. Jpn* **94**, 357–376.

[bb20] Kim, J. E., Han, T. H., Lee, S. H., Kim, J. Y., Ahn, C. W., Yun, J. M. & Kim, S. O. (2011). *Angew. Chem. Int. Ed.* **50**, 3043–3047. 10.1002/anie.201004692 21404395

[bb23] Kurta, R. P., Wiegart, L., Fluerasu, A. & Madsen, A. (2019). *IUCrJ* **6**, 635–648. 10.1107/S2052252519005499 PMC6608627 31316808

[bb25] Kusaka, Y., Hasegawa, T. & Kaji, H. (2019). *J. Phys. Chem. A* **123**, 10333–10338. 10.1021/acs.jpca.9b04437 31682439

[bb26] Lama, H., Yamamoto, M. J., Furuta, Y., Shimaya, T. & Takeuchi, K. A. (2024). *Proc. Natl Acad. Sci. USA Nexus* **3**, pgae238. 10.1093/pnasnexus/pgae238 PMC11238424 38994498

[bb27] Lapkin, D., Shabalin, A., Meijer, J.-M., Kurta, R., Sprung, M., Petukhov, A. V. & Vartanyants, I. A. (2022). *IUCrJ* **9**, 425–438. 10.1107/S2052252522004250 PMC9252153 35844483

[bb28] Lehmkühler, F., Grübel, G. & Gutt, C. (2014). *J. Appl. Cryst.* **47**, 1315–1323.

[bb29] Lehmkuhler, F., Hankiewicz, B., Schroer, M. A., Muller, L., Ruta, B., Sheyfer, D., Sprung, M., Tono, K., Katayama, T., Yabashi, M., Ishikawa, T., Gutt, C. & Grubel, G. (2020). *Sci. Adv.* **6**, eabc5916. 10.1126/sciadv.abc5916 PMC7577711 33087351

[bb30] Letz, M., Schilling, R. & Latz, A. (2000). *Phys. Rev. E* **62**, 5173–5178. 10.1103/physreve.62.5173 11089079

[bb32] Liang, J., Feng, X., Zheng, N., Wang, H., Ni, R. & Zhang, Z. (2025). *Phys. Rev. Lett.* **134**, 038202. 10.1103/PhysRevLett.134.038202 39927969

[bb34] Lopes, J. T., Romano, F., Grelet, E., Franco, L. F. M. & Giacometti, A. (2021). *J. Chem. Phys.* **154**, 104902. 10.1063/5.0040942 33722037

[bb35] Malmerberg, E., Kerfeld, C. A. & Zwart, P. H. (2015). *IUCrJ* **2**, 309–316. 10.1107/S2052252515002535 PMC4420540 25995839

[bb36] Matos, C. R., Xavier, M. J., Barreto, L. S., Costa, N. B. Jr & Gimenez, I. F. (2007). *Anal. Chem.* **79**, 2091–2095. 10.1021/ac061991n 17326603

[bb37] Mishra, C. K. & Ganapathy, R. (2015). *Phys. Rev. Lett.* **114**, 198302. 10.1103/PhysRevLett.114.198302 26024202

[bb38] Mishra, C. K., Hima Nagamanasa, K., Ganapathy, R., Sood, A. K. & Gokhale, S. (2014). *Proc. Natl Acad. Sci. USA* **111**, 15362–15367. 10.1073/pnas.1413384111 PMC4217396 25313030

[bb39] Mishra, C. K., Rangarajan, A. & Ganapathy, R. (2013). *Phys. Rev. Lett.* **110**, 188301. 10.1103/PhysRevLett.110.188301 23683247

[bb40] Miyamoto, N. & Nakato, T. (2002). *Adv. Mater.* **14**, 1267–1270.

[bb41] Nakayama, M., Kajiyama, S., Kumamoto, A., Nishimura, T., Ikuhara, Y., Yamato, M. & Kato, T. (2018). *Nat. Commun.* **9**, 568. 10.1038/s41467-018-02932-7 PMC5805687 29422609

[bb42] Nakayama, M. & Kato, T. (2022). *Acc. Chem. Res.* **55**, 1796–1808. 10.1021/acs.accounts.2c00063 PMC9260960 35699654

[bb43] Odriozola, G. (2012). *J. Chem. Phys.* **136**, 134505. 10.1063/1.3699331 22482570

[bb44] Pal, A., Kamal, M. A., Holmqvist, P. & Schurtenberger, P. (2021). *Phys. Rev. Res.* **3**, 023254.

[bb45] Pal, A., Kamal, M. A. & Schurtenberger, P. (2022). *J. Colloid Interface Sci.* **621**, 352–359. 10.1016/j.jcis.2022.04.063 35468558

[bb46] Pal, A., Martinez, V. A., Ito, T. H., Arlt, J., Crassous, J. J., Poon, W. C. K. & Schurtenberger, P. (2020). *Sci. Adv.* **6**, eaaw9733. 10.1126/sciadv.aaw9733 PMC6968932 32010765

[bb47] Pande, K., Donatelli, J. J., Malmerberg, E., Foucar, L., Bostedt, C., Schlichting, I. & Zwart, P. H. (2018). *Proc. Natl Acad. Sci. USA* **115**, 11772–11777. 10.1073/pnas.1812064115 PMC6243272 30373827

[bb48] Poulos, A. S., Constantin, D., Davidson, P., Pansu, B., Freyssingeas, E., Madsen, A. & Chané*ac*, C. (2010). *J. Chem. Phys.* **132**, 091101. 10.1063/1.3330920 20210381

[bb49] Roller, J., Geiger, J. D., Voggenreiter, M., Meijer, J. M. & Zumbusch, A. (2020). *Soft Matter* **16**, 1021–1028. 10.1039/c9sm01926j 31854439

[bb50] Roller, J., Laganapan, A., Meijer, J. M., Fuchs, M. & Zumbusch, A. (2021). *Proc. Natl Acad. Sci. USA* **118**, e2018072118. 10.1073/pnas.2018072118 PMC7826331 33397813

[bb52] Schroer, M. A., Gutt, C., Lehmkühler, F., Fischer, B., Steinke, I., Westermeier, F., Sprung, M. & Grübel, G. (2015). *Soft Matter* **11**, 5465–5472. 10.1039/c5sm00609k 26061482

[bb53] Segovia-Gutiérrez, J. P., Escobedo-Sánchez, M. A., Sarmiento-Gómez, E. & Egelhaaf, S. U. (2020). *Front. Phys.* **7**, 224.

[bb54] Solomon, M. J. & Spicer, P. T. (2010). *Soft Matter* **6**, 1391–1400.

[bb55] Sprakel, J., van der Gucht, J., Cohen Stuart, M. A. & Besseling, N. A. (2007). *Phys. Rev. Lett.* **99**, 208301. 10.1103/PhysRevLett.99.208301 18233191

[bb56] Striker, N. N., Lokteva, I., Dartsch, M., Dallari, F., Goy, C., Westermeier, F., Markmann, V., Hövelmann, S. C., Grübel, G. & Lehmkühler, F. (2023). *J. Phys. Chem. Lett.* **14**, 4719–4725. 10.1021/acs.jpclett.3c00631 PMC10226114 37171882

[bb57] Takae, K. & Onuki, A. (2014). *Phys. Rev. E* **89**, 022308. 10.1103/PhysRevE.89.022308 25353473

[bb58] Tamasaku, K., Tanaka, Y., Yabashi, M., Yamazaki, H., Kawamura, N., Suzuki, M. & Ishikawa, T. (2001). *Nucl. Instrum. Methods Phys. Res. A* **467–468**, 686–689.

[bb59] van der Kooij, F. M. & Lekkerkerker, H. N. W. (1998). *J. Phys. Chem. B* **102**, 7829–7832.

[bb60] Vroege, G. J. & Lekkerkerker, H. N. W. (1992). *Rep. Prog. Phys.* **55**, 1241–1309.

[bb61] Wochner, P., Gutt, C., Autenrieth, T., Demmer, T., Bugaev, V., Ortiz, A. D., Duri, A., Zontone, F., Grübel, G. & Dosch, H. (2009). *Proc. Natl Acad. Sci. USA* **106**, 11511–11514. 10.1073/pnas.0905337106 PMC2703671 20716512

[bb62] Xu, Z. & Gao, C. (2011). *Nat. Commun.* **2**, 571. 10.1038/ncomms1583 PMC3247827 22146390

[bb63] Zaluzhnyy, I. A., Kurta, R. P., Scheele, M., Schreiber, F., Ostrovskii, B. I. & Vartanyants, I. A. (2019). *Materials* **12**, 3436. 10.3390/ma12213464 PMC6862311 31652689

[bb64] Zhao, W., Miyashita, O., Nakano, M. & Tama, F. (2024). *IUCrJ* **11**, 92–108. 10.1107/S2052252523009831 PMC10833384 38096036

[bb65] Zheng, Z., Ni, R., Wang, F., Dijkstra, M., Wang, Y. & Han, Y. (2014). *Nat. Commun.* **5**, 3829. 10.1038/ncomms4829 PMC4024749 24807069

[bb66] Zheng, Z., Wang, F. & Han, Y. (2011). *Phys. Rev. Lett.* **107**, 065702. 10.1103/PhysRevLett.107.065702 21902341

