# Supplementary material for: Time-resolved coherent X-ray scattering analysis of nanorod colloids: dynamics and photon-statistical effects in angular X-ray cross-correlation analysis
Source: J Synchrotron Radiat. 2026 Oct 27;33(Pt 6):1769–78. doi: 10.1107/S160057752600980X (PMC13753526; doi:10.1107/S160057752600980X)
Supplement: Supplementary file 1 [file s-33-01769-sup1.pdf]

JOURNAL OF SYNCHROTRON RADIATION

Volume 33 (2026)

Supporting information for article:

Time-resolved coherent X-ray scattering analysis of nanorod colloids: dynamics and photon-statistical effects in angular X-ray cross-correlation analysis

Taiki Hoshino, Yoshifumi Amamoto, Takahiro Mikami, Yoshiaki Honjo, Kyosuke Ozaki, Toshiyuki Nishiyama Hiraki, Haruki Nishino, Yasumasa Joti, Takaki Hatsui, Yoshiki Kohmura and Takashi Kato

Contents

1. **Fig. S1** Polarized optical microscope images at Φ=0.24 - 0.26.
2. **Fig. S2** SAXS profile of the dilute dispersion.
3. **Fig. S3** $S(q_\perp)$ obtained at Φ = 0.25 for *Z* = 0-1 mm.
4. **Fig. S4** Intensity autocorrelation functions $g_2(q, t)$ used to estimate the speckle contrast.
5. **Fig. S5** q dependence of γ for various Φ.
6. **Fig. S6** q dependence of Γq^{-2} for various Φ.
7. **Fig. S7** *Z* dependence of Γ_p at $\Phi = 0.25$.
8. **Fig. S8** Distributions of p_1 at 11 different positions between Z = 0 and 1 mm.
9. **Fig. S9** Comparison of the frame-averaged AXCCF with the AXCCF from the time-averaged intensity.
10. **Fig. S10** Average azimuthal profiles obtained from the experimental and simulated data.
11. **Fig. S11** Single-frame azimuthal profiles before and after photon quantization.
12. **Fig. S12** Distributions of p_1 for the quantized experimental and simulated data.
13. **Fig. S13** Reference azimuthal intensity profile for the photon-count-dependence simulations.
14. **Fig. S14** Temporal correlation function of $C_q(\delta\phi)$.

Comparison with Fourier analysis and the orientational order parameter

15. **Fig. S15** Normalized Fourier power p_n of the single-frame intensity profiles.
16. **Fig. S16** Correlation between p_2 and the shifted PC1 score.
17. **Fig. S17** Dependence of σ_S on the number of accumulated frames.

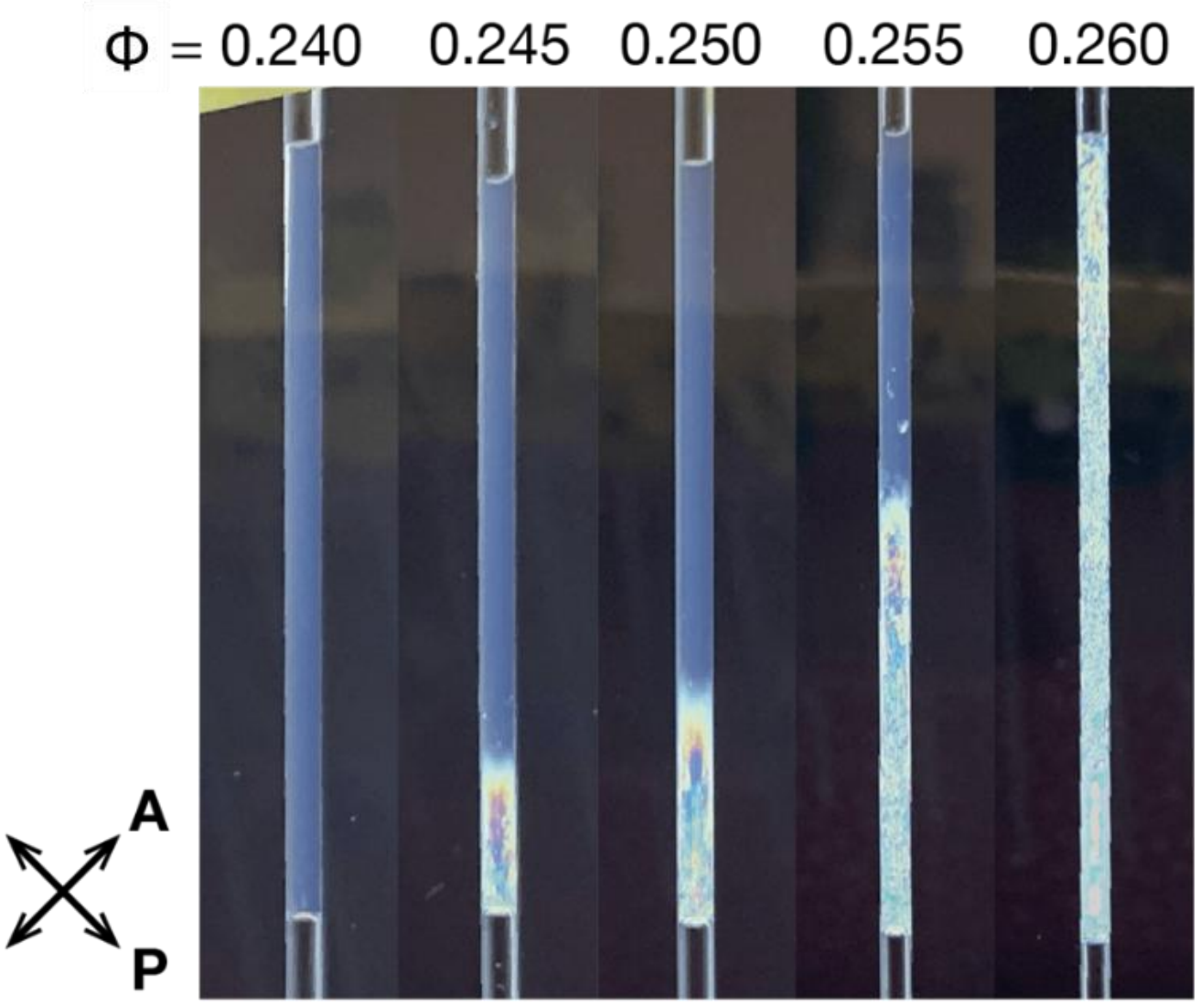

Figure S1 Polarized optical microscopy (POM) images for the nanorod colloidal particles at volume fraction of Φ = 0.24 – 0.26, taken three weeks after sealing. A: analyzer. P: polarizer.

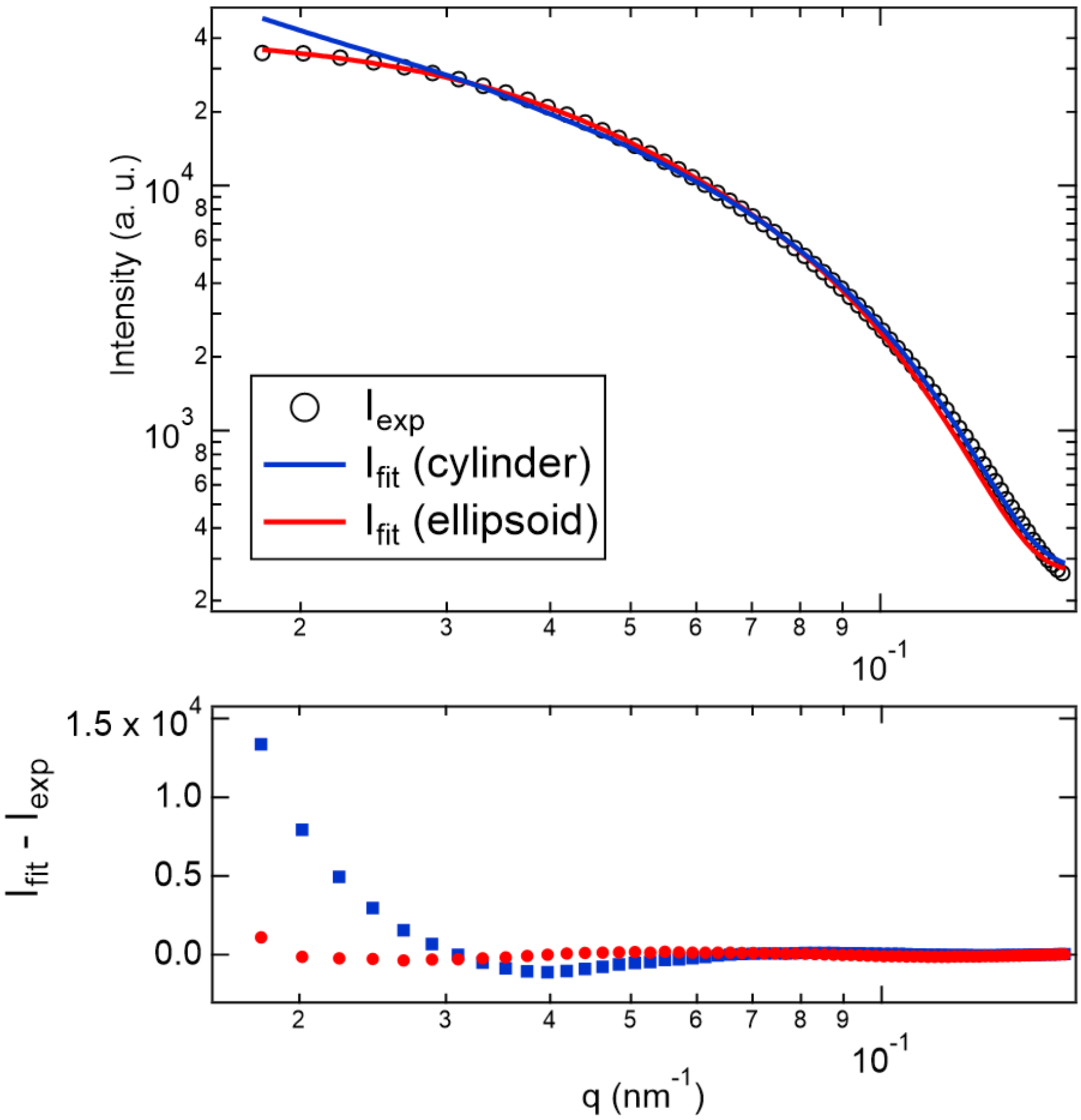

Figure S2 (a) SAXS profile of the dilute dispersion at Φ = 0.005. The blue line represents the scattering from randomly oriented cylinders, and the red line represents the fitting curve obtained using the scattering function of randomly oriented ellipsoids. (b) Residuals between the experimental data and the fitting curves. The ellipsoidal model reproduces the experimental data better than the cylindrical model. The obtained dimensions (50 nm in diameter along the short axis and 157 nm in length along the long axis) are consistent with electron microscopy results.

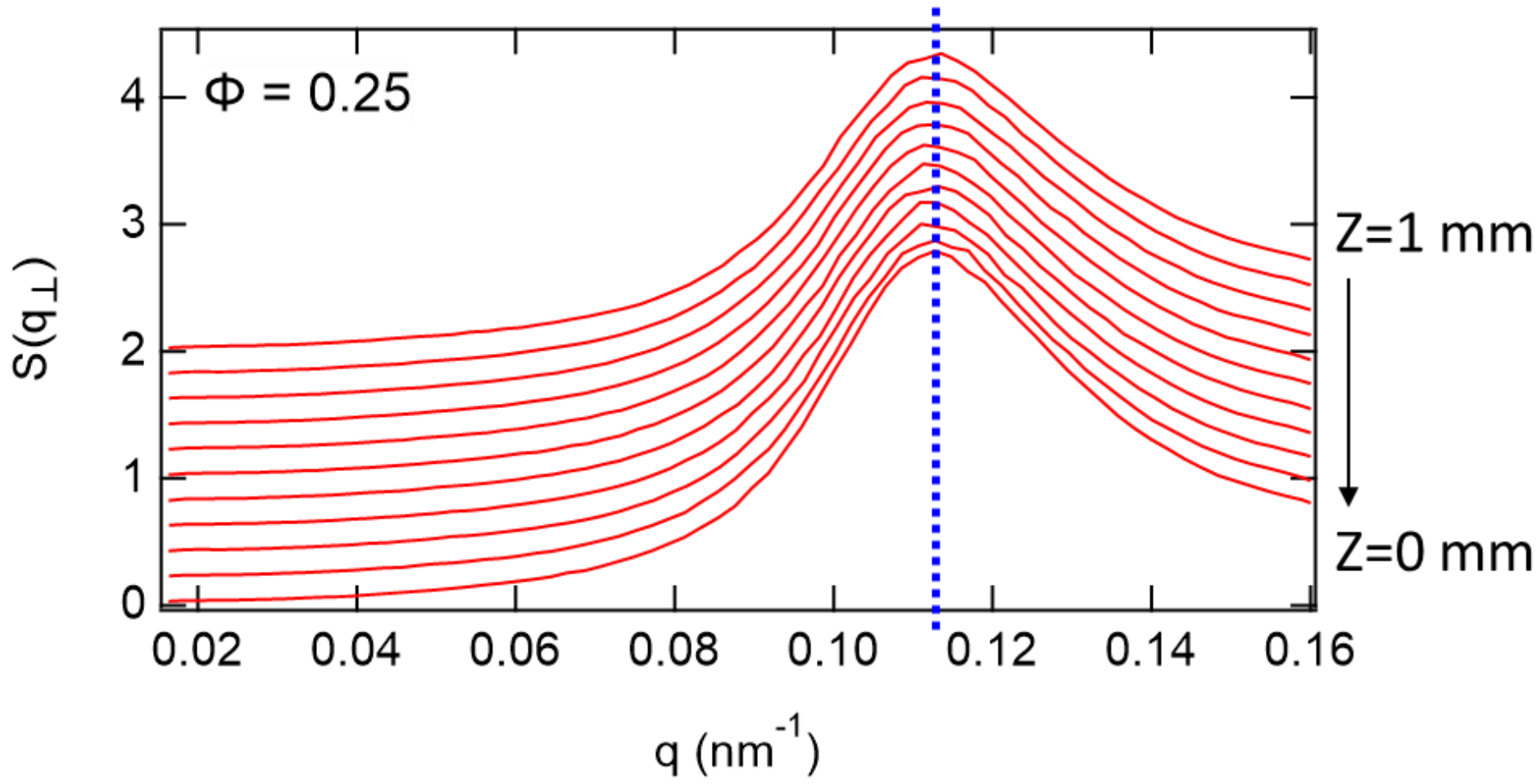

Figure S3 $S(q_\perp)$ obtained at Φ = 0.25 for *Z* = 0-1 mm. The *Z* dependence was negligible small.

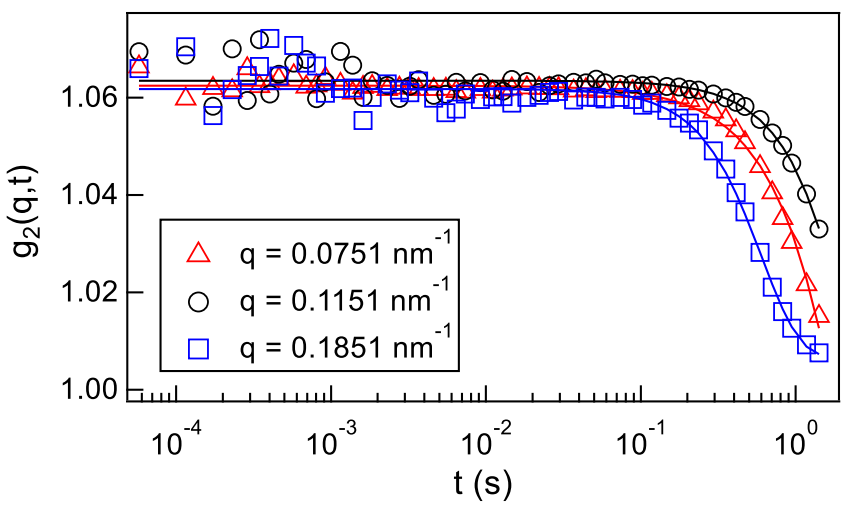

Figure S4 Intensity autocorrelation functions $g_2(q, t)$ obtained from an additional concentrated nanorod dispersion ($\Phi = 0.31$) measured 11 days after sample preparation under the same experimental configuration as that used in the present study. The slow relaxation of this concentrated sample allowed the speckle contrast to be estimated from the short-time amplitude of $g_2 - 1$, yielding $\beta \simeq 0.06$.

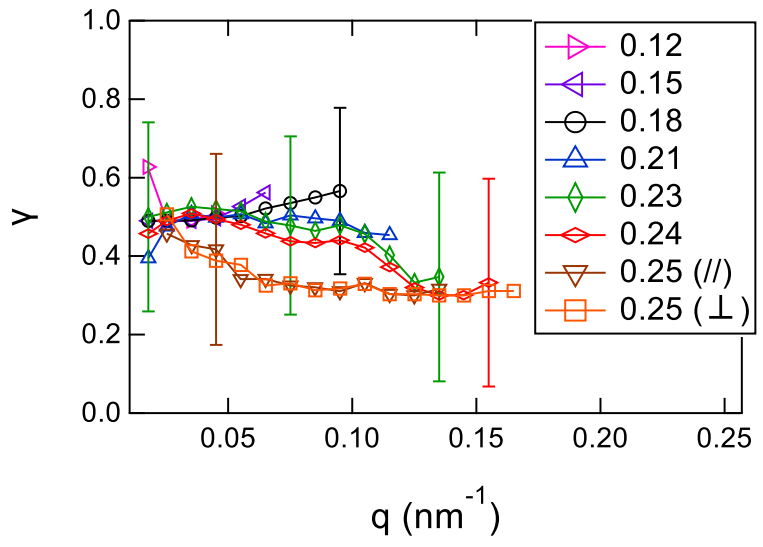

Figure S5 q dependence of γ for various Φ. To avoid overcrowding, error bars are shown only for representative points.

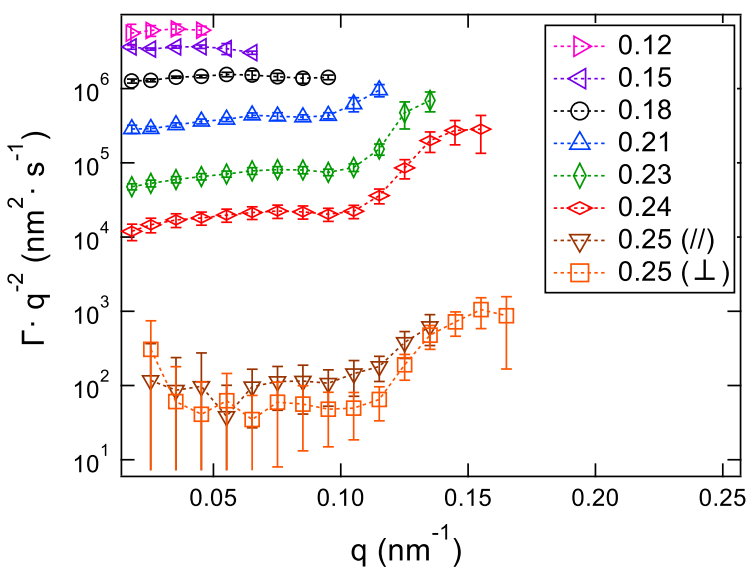

Figure S6 q dependence of Γq^{-2}. Γ tends to increase with q, indicating that $\Gamma \propto q^n$ with $n > 2$.

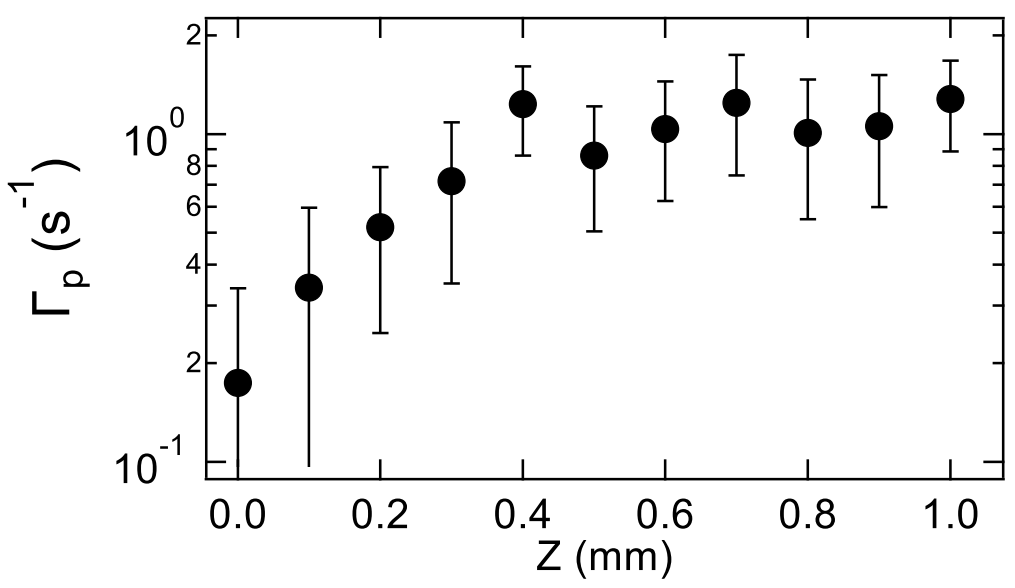

Figure S7 *Z* dependence of Γ_p at $\Phi = 0.25$.

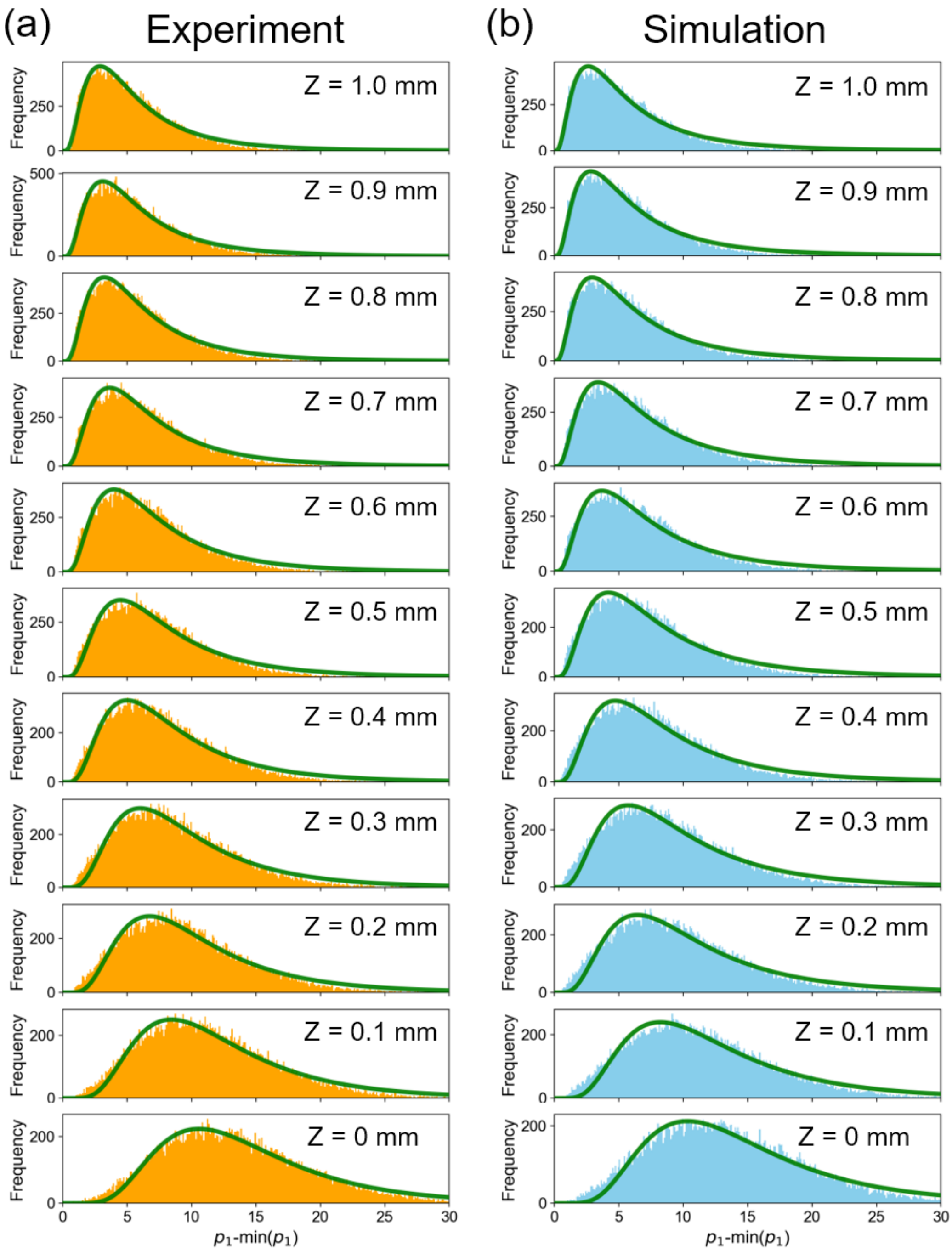

Figure S8 Distributions of p_1 at 11 different positions between Z = 0 and 1 mm for experimental data (a) and simulated data (b).

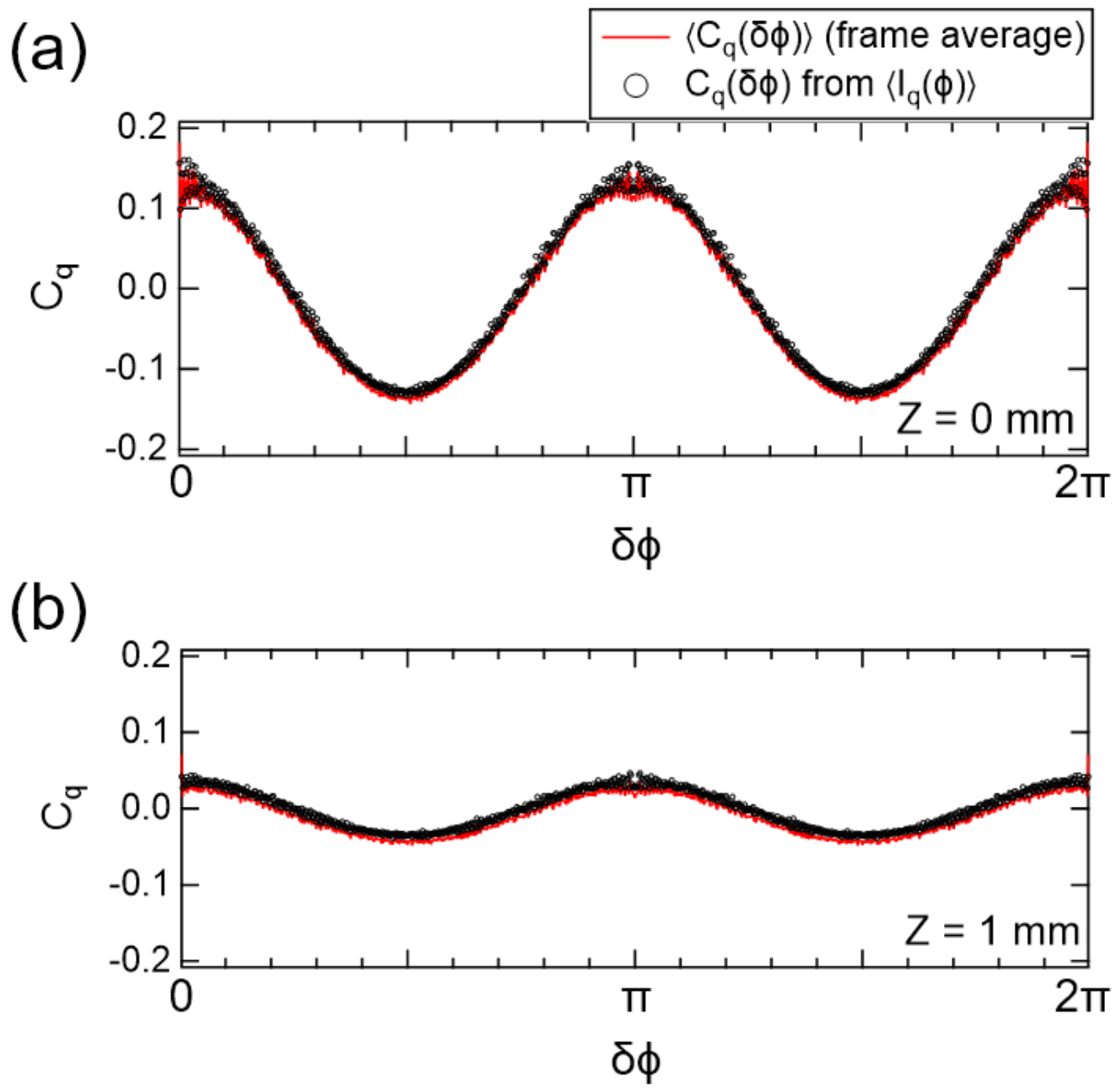

Figure S9 Comparison of the frame-averaged AXCCF, $\langle C_q(\delta\phi)\rangle$ (red lines), with the AXCCF calculated from the time-averaged azimuthal scattering intensity, $C_q[\langle I_q(\phi)\rangle]$ (open circles), at (a) $Z = 0$mm and (b) $Z = 1$mm for $\Phi = 0.25$at $q = 0.1130$nm^{-1}. The two results show close agreement over most of the angular range.

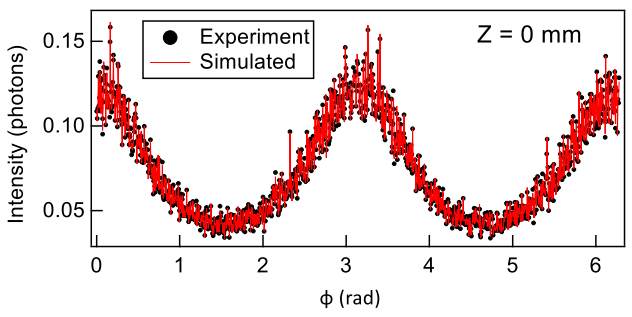

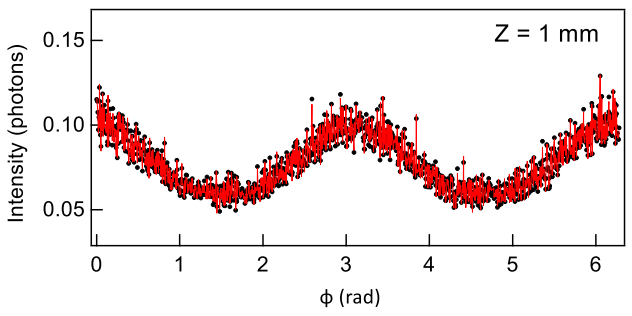

Figure S10 Comparison of the average azimuthal profiles obtained from the experimental and simulated data. The good agreement between the two confirms that the simulated dataset reproduces the mean experimental scattering anisotropy.

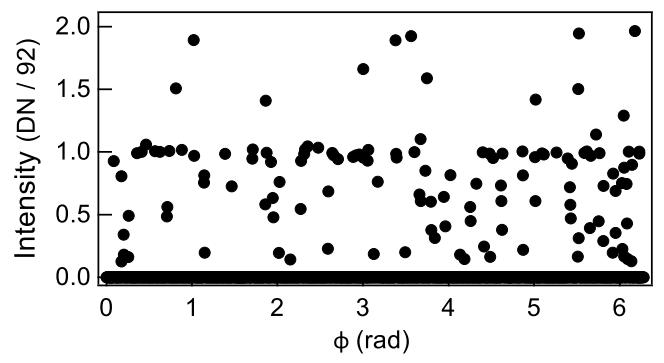

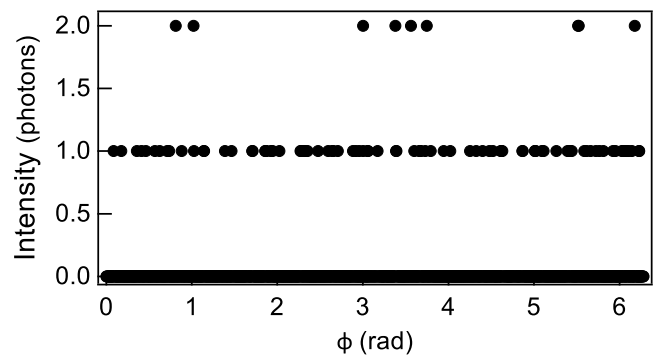

Figure S11 Representative single-frame azimuthal profiles obtained from the raw detector data (top) and after quantization using 92 DN per photon (bottom).

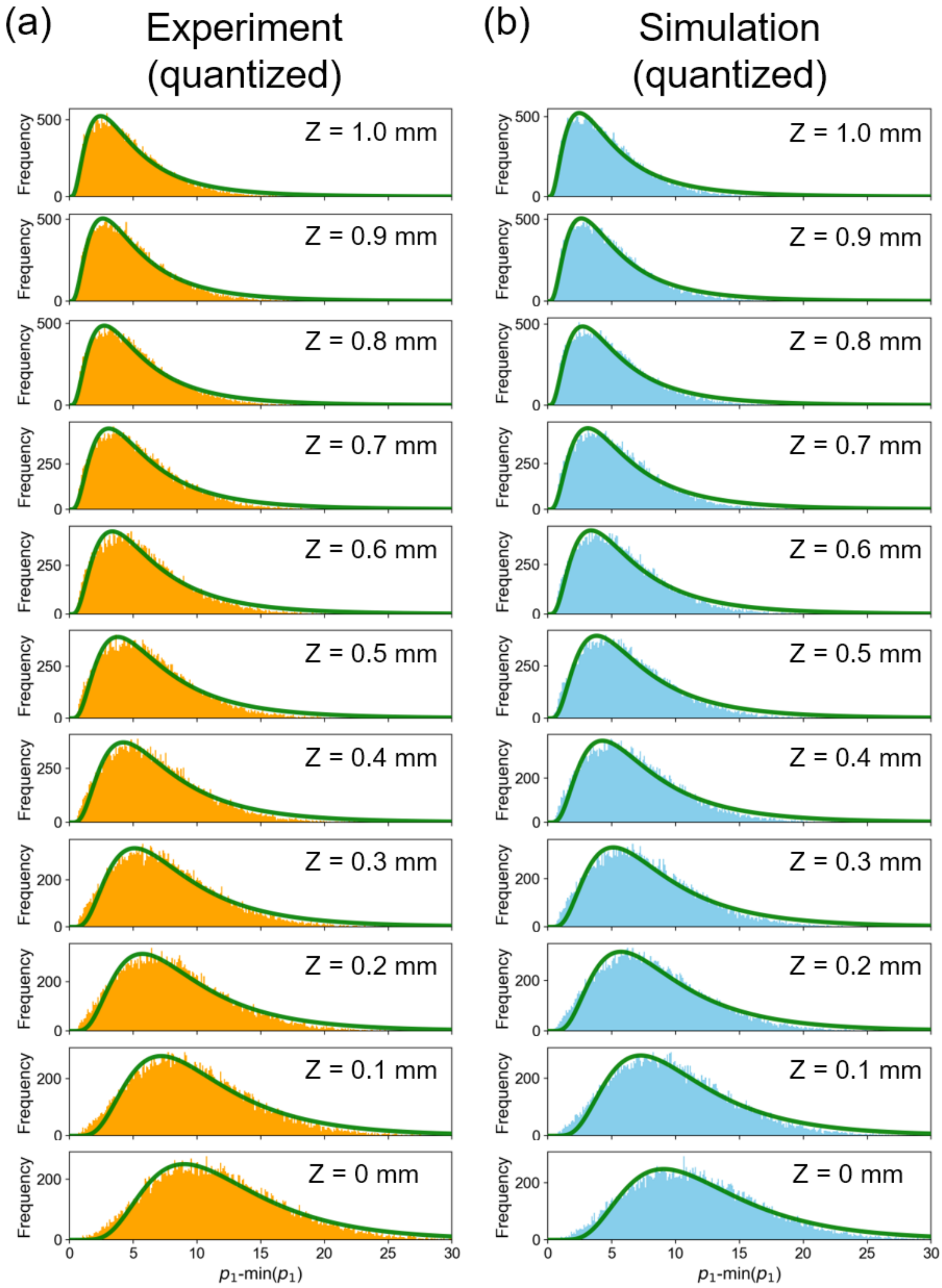

Figure S12 Distributions of p_1 at 11 different positions between Z = 0 and 1 mm for quantized experimental data (a) and the corresponding simulated data (b).

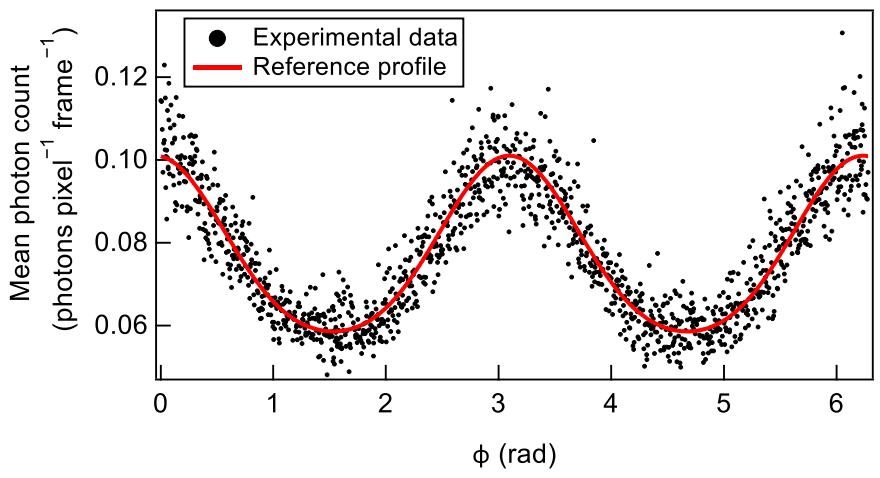

Figure S13 Reference azimuthal intensity profile used for the photon-count-dependence simulations shown in Fig. 10. The black circles show the all-frame integrated experimental azimuthal profile at $Z = 1$mm. The red line shows the reference profile reconstructed using the Fourier model

$$I_{\mathrm{ref}}(\phi) = a_0 + a_{2c}\cos(2\phi) + a_{2s}\sin(2\phi) + a_{4c}\cos(4\phi) + a_{4s}\sin(4\phi),$$

where a_0, a_{2c}, a_{2s}, a_{4c}, and $a_{4s}$are the fitted coefficients. The reference profile was used as the mean angular intensity distribution in the simulations, with its shape fixed while the mean photon count per pixel per frame was varied.

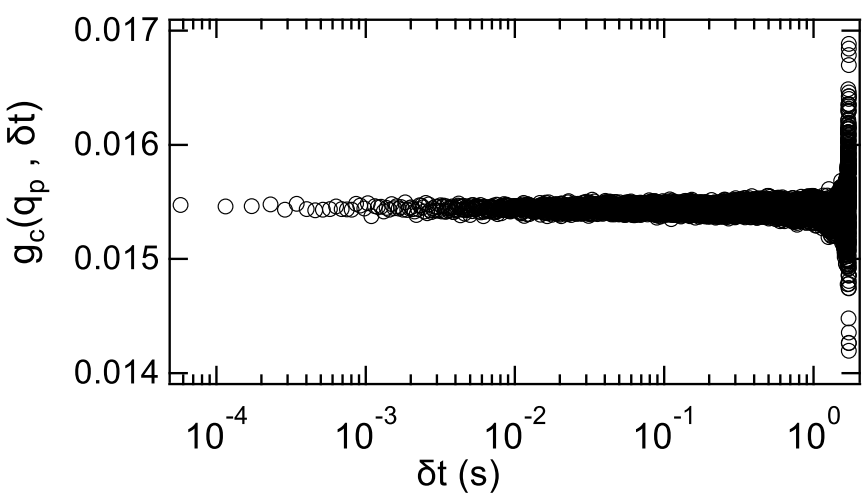

Figure S14 Temporal correlation function of $C_q(\delta\phi)$ at Φ = 0.25, *Z*=0.6 mm, for a representative example showing no significant relaxation behavior.

Comparison with Fourier analysis and the orientational order parameter

To verify the angular-correlation features extracted by AXCCF-PCA using an independent approach, we performed Fourier analysis of the scattering intensity in each individual frame. In general, the Fourier components of an angular correlation function are closely related to the powers of the Fourier components of the azimuthal intensity distribution $I(q, \phi)$. Therefore, for the same q region used in the AXCCF analysis ($q \simeq 0.113\ \mathrm{nm}^{-1}$), we calculated the Fourier coefficient of order n, I_n, for each frame. Here, n is the integer Fourier order, I_n is the n th complex Fourier coefficient of $I(q, \phi)$, and I_0 is the zeroth-order coefficient, corresponding to the azimuthally averaged intensity. The normalized Fourier power was then evaluated as $p_n = |I_n|^2/|I_0|^2$.

Fig. S15 shows the normalized Fourier powers obtained for $\Phi = 0.25$ at $Z = 0\ \mathrm{mm}$ and $Z = 1\ \mathrm{mm}$. At both positions, the $n = 2$ component is substantially larger than the components of other orders, confirming that the dominant angular anisotropy in the single-frame scattering

intensity is characterized by twofold symmetry. This result is consistent with the first principal component obtained from PCA of the AXCCFs being dominated by a component with a period of π.

We further examined, on a frame-by-frame basis, the relationship between the normalized power of the second-order Fourier component p_2 and the PC1 score obtained from the AXCCF (**Fig. S16**). A strong positive correlation was observed, with Pearson correlation coefficients of 0.944 at $Z = 0\,\mathrm{mm}$ and 0.937 at $Z = 1\,\mathrm{mm}$. This result shows that the dominant mode of variation extracted by AXCCF-PCA corresponds mainly to the strength of the twofold-symmetric component of the scattering intensity. Thus, the dominant twofold symmetry in these data can also be evaluated directly by Fourier analysis. However, AXCCF–PCA extracts the dominant pattern of frame-to-frame variation from the entire angular correlation profile in a data-driven manner; therefore, we used AXCCF–PCA as the primary analysis in this study.

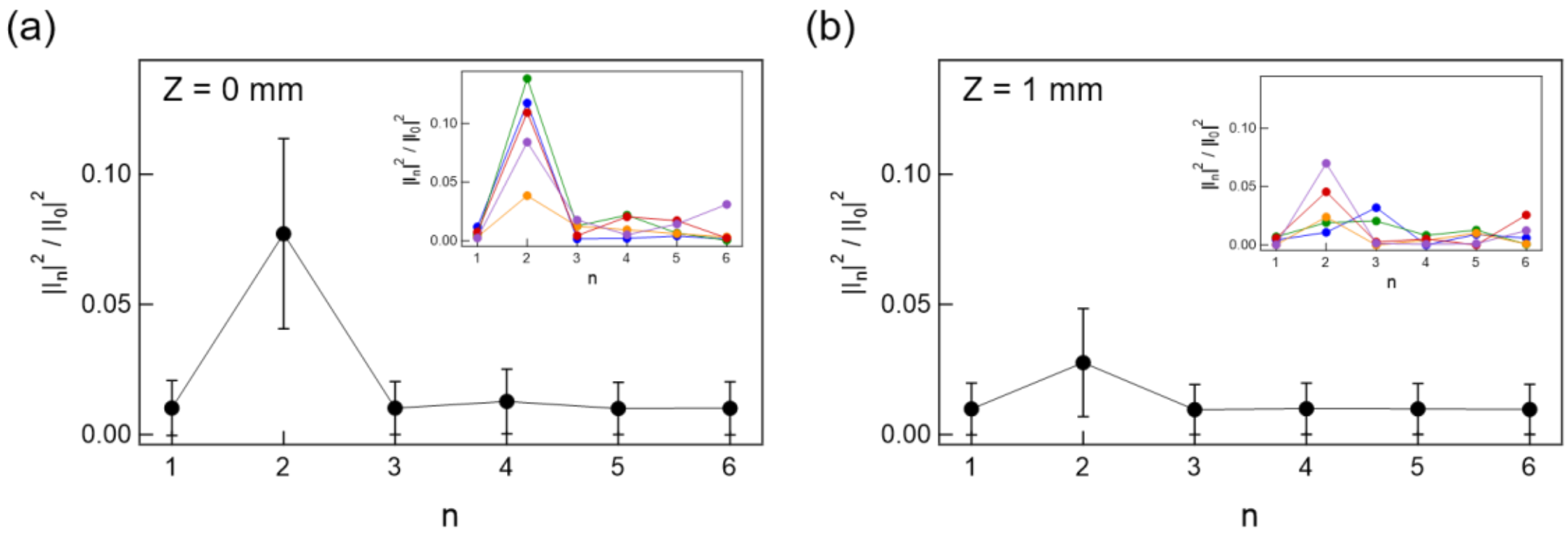

Figure S15 Normalized Fourier power, p_n, calculated from the single-frame azimuthal intensity profiles at $q \simeq 0.113\,\mathrm{nm}^{-1}$ for $\Phi = 0.25$ at (a) $Z = 0\,\mathrm{mm}$ and (b) $Z = 1\,\mathrm{mm}$. The black circles and error bars represent the mean and standard deviation over all frames, respectively. The insets show representative results for five individual frames. At both positions, the $n = 2$ component is dominant over the other Fourier components.

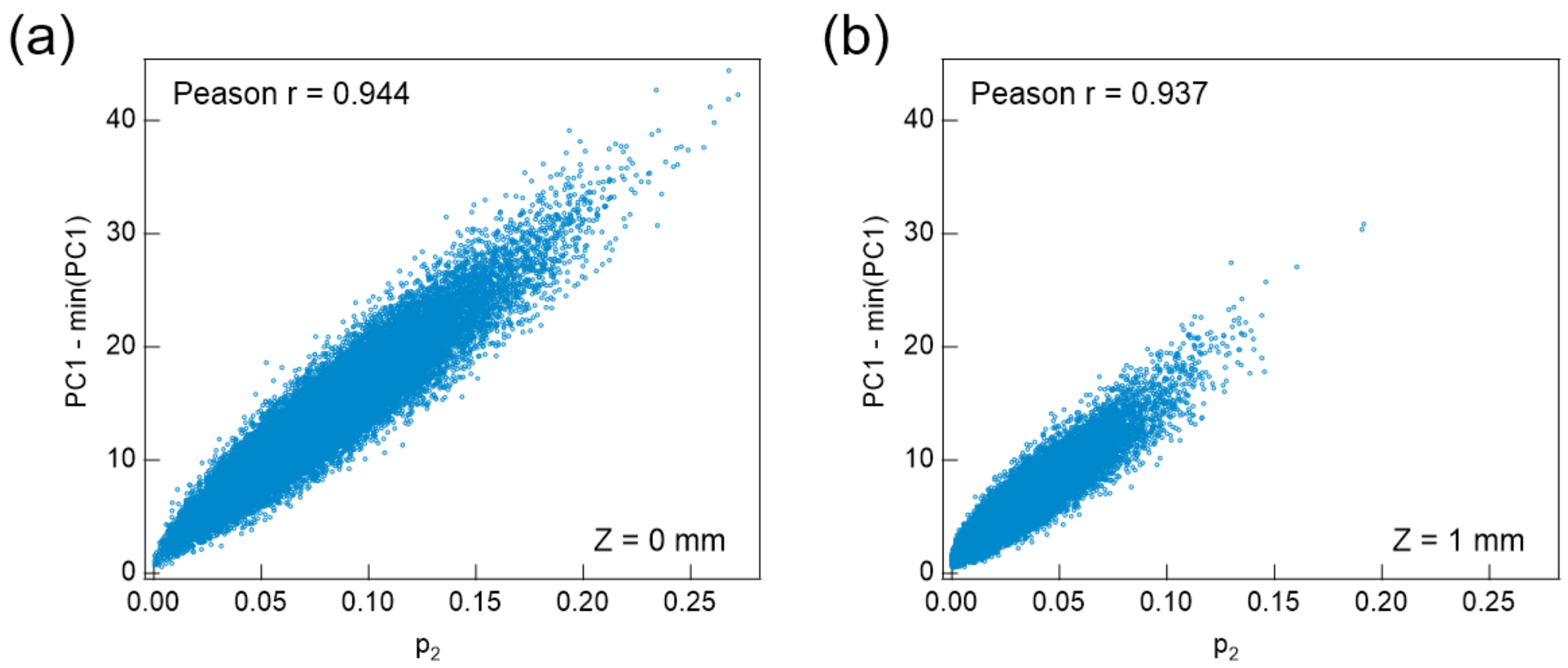

Figure S16 Correlation between p_2 and the shifted PC1 score, $\mathrm{PC1} - \min(\mathrm{PC1})$, calculated for each frame at $q \simeq 0.113\,\mathrm{nm}^{-1}$ for $\Phi = 0.25$ at (a) $Z = 0\,\mathrm{mm}$ and (b) $Z = 1\,\mathrm{mm}$. Each point represents one frame. The Pearson correlation coefficients are 0.944 and 0.937 for $Z = 0\,\mathrm{mm}$ and $Z = 1\,\mathrm{mm}$, respectively.

We also examined whether accumulating multiple consecutive frames improves the statistical precision of the orientational order parameter S obtained from the azimuthal intensity distribution. After summing non-overlapping groups of N_{acc} consecutive frames, S was calculated from each accumulated image using the same procedure as in Eq. (1) of the main text. **Fig. S17** shows the dependence of the standard deviation σ_S on N_{acc} at $Z = 0$ mm and $Z = 1$ mm. σ_S clearly decreases with increasing N_{acc} and, at relatively small N_{acc}, exhibits a trend close to the $N_{acc}^{-1/2}$ dependence expected for independent statistical fluctuations. Thus, accumulating multiple frames reduces the statistical variation in S. However, this improvement is accompanied by a loss of temporal resolution, and the trade-off between statistical precision and temporal resolution must therefore be considered when evaluating rapid temporal changes.

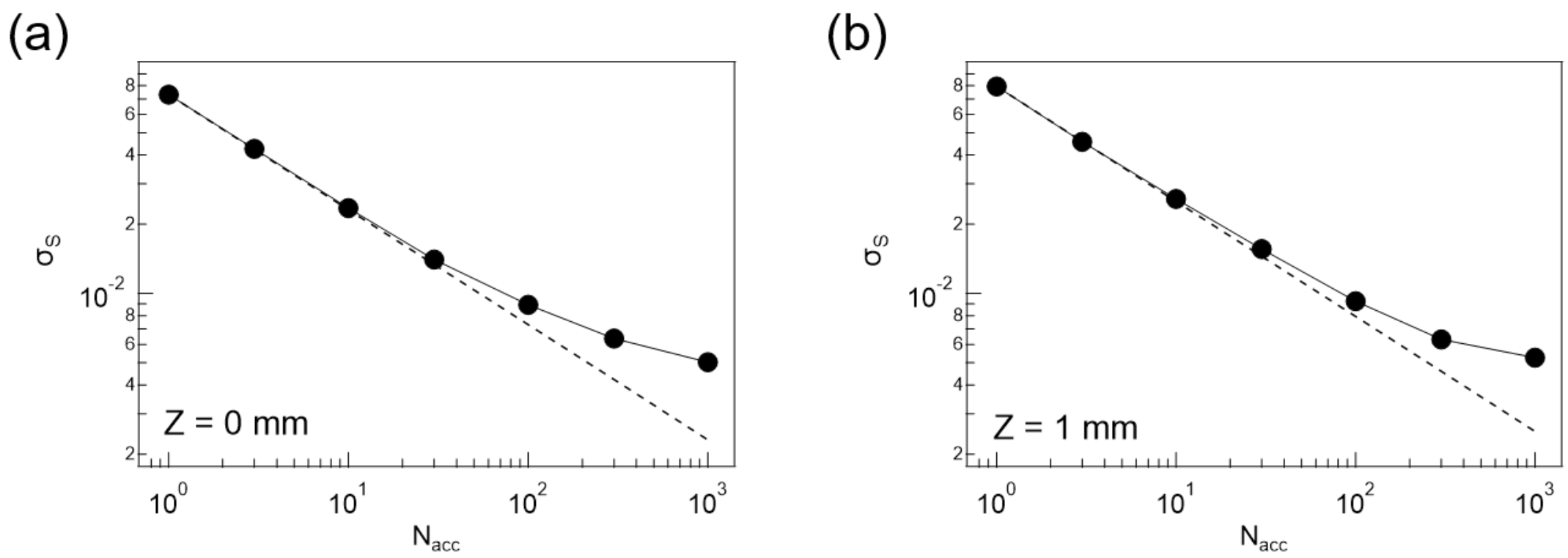

Figure S17 Dependence of the standard deviation σ_S of S on the number N_{acc} of consecutively accumulated frames for $\Phi = 0.25$ at (a) $Z = 0$ mm and (b) $Z = 1$ mm. The frames were divided into non-overlapping groups of N_{acc} consecutive frames, and S was calculated from each accumulated image using the same procedure as in Eq. (1). The dashed lines indicate the $N_{acc}^{-1/2}$ dependence expected for independent statistical fluctuations.
